# What evidence supports primary prevention strategies for sport-related concussion? An umbrella review

**DOI:** 10.5114/biolsport.2026.161108

**Published:** 2026-07-13

**Authors:** Khaled Trabelsi, Omar Boukhris, Souhail Chebbi, Waqar Husain, Achraf Ammar, Louis Holtzhausen, Hamdi Chtourou, Karim Chamari, Haitham Jahrami

**Affiliations:** 1 Research laboratory Education, Motricité, Sport et Santé, EM2S, LR19JS01, High Institute of Sport and Physical Education of Sfax, University of Sfax, Tunisia; 2 Department of Movement Sciences and Sports Training, School of Sport Science, The University of Jordan, Amman, Jordan; 3 Sport, Performance, and Nutrition Research Group, School of Allied Health, Human Services and Sport, La Trobe University, Melbourne, Australia; 4 SIESTA Research Group, School of Allied Health, Human Services and Sport, La Trobe University, Melbourne, Australia; 5 Aspetar Orthopedic and Sports Medicine Hospital, Doha, Qatar; 6 Department of Humanities, COMSATS University Islamabad, Islamabad Campus, Park Road, Islamabad, Pakistan; 7 Department of Training and Movement Science, Institute of Sport Science, Johannes Gutenberg-University Mainz, Mainz, Germany; 8 Research Laboratory, Molecular Bases of Human Pathology, LR19ES13, Faculty of Medicine of Sfax, University of Sfax, Sfax 3000, Tunisia; 9 Nutrition and Food Technology Department, School of Agriculture, the University of Jordan, Amman, Jordan; 10 Section Sports Medicine, University of Pretoria, South Africa; 11 Department of Exercise and Sport Sciences, University of the Free State, South Africa; 12 High Institute of Sport and Physical Education, University of Sfax, Sfax, Tunisia; 13 Research Unit Physical Activity, Sport and Health, UR18JS01, National Observatory of Sport, Tunis, Tunisia; 14 Naufar Center, Doha, Qatar; 15 Higher institute of Sport and Physical Education, ISSEP Ksar Saïd, Manouba University, Tunis, Tunisia; 16 Department of Psychiatry, College of Medicine and Health Sciences, Arabian Gulf University, Manama, Bahrain; 17 Government Hospitals, Manama, Bahrain

**Keywords:** Contact sport, Head protective devices, Muscle strength, Risk reduction, Traumatic brain injury, Overview of systematic reviews

## Abstract

This umbrella review aimed to systematically evaluate and synthesize evidence from published systematic reviews on the effectiveness of primary prevention strategies for sport-related concussion (SRC) in athletes, and to identify areas of uncertainty relevant to policy and practice. A comprehensive search of the Cochrane Database of Systematic Reviews, Epistemonikos, ERIC, PsycINFO, PubMed, Scopus, SPORTDiscus, and Web of Science was conducted from 2001 to April 2025, with an updated search in November 2025. Peerreviewed systematic reviews, with or without meta-analyses, assessing primary prevention strategies to reduce SRC incidence in competitive athletes of any age or sex were eligible. Interventions included rule or policy changes, protective equipment, educational programs and training-based strategies. Twenty-five systematic reviews were included, eight of which contained meta-analyses; all were rated as critically low quality using the AMSTAR-2 tool. Rule and policy changes, particularly the disallowance of body checking in youth ice hockey, demonstrated the most consistent evidence for reducing SRC incidence. Meta-analyses evaluating mouthguards and headgear showed substantial overlap of primary studies and inconsistent effects. Educational interventions and training-based programs, including structured warm-up programs such as FIFA 11+ Kids, produced inconclusive findings. Evidence specific to female athletes was limited and insufficient to support any particular prevention strategy. Overall, rule and policy changes that reduce exposure to high-risk contact currently have the strongest supporting evidence for SRC prevention, whereas evidence for equipment, education and training remains mixed. Given the critically low methodological quality of existing reviews, higher-quality evidence syntheses and robust primary studies are urgently needed.

## INTRODUCTION

The recent Consensus Statement on Concussion in Sport defines sport-related concussion (SRC) as a traumatic brain injury caused by external forces applied to the head, neck, or body, resulting in force transmission to the brain during sport- or exercise-related activities [1]. SRCs are among the most common and complex injuries encountered in contact and collision sports [2]. Although recovery typically occurs within weeks, some individuals may experience symptoms lasting longer than one month [3]. SRC is associated with a wide range of clinical symptoms and signs, which may or may not involve loss of consciousness [1, 4]. Typical manifestations include headache, cognitive slowing, irritability, amnesia, difficulty concentrating, nausea, vomiting, and sleep disturbances [1, 4, 5]. Concussion can delay an athlete’s return to sport [6], not only because of physical symptoms but also because of psychosocial factors such as fear of reinjury [7], social expectations [8], and disruptions in selfidentity [7, 8]. Moreover, repeated concussions may increase the risk of neurodegenerative diseases in late adulthood [9, 10], and in some cases, can be fatal [11]. SRC imposes substantial financial burdens on athletes, insurance providers, and healthcare systems because of rising entitlement claims, reduced or lost earnings, and long-term medical costs [12–15].

Recent epidemiological evidence has revealed the highest SRC incidence rates per 1,000 athlete exposures in taekwondo, rugby union, and ice hockey, and per 1,000 player hours in rugby sevens, rugby league, and rugby union [16]. Furthermore, an overview of systematic reviews reported that the incidence of SRC appears to be highest in rugby, snowboarding, ice hockey, and American football, with rates that are consistently greater during games than during practices [17]. Moreover, the incidence of SRC is greater in amateurs than in professional rugby athletes, and female athletes experience higher SRC rates than males do in baseball-softball, basketball, and soccer [17]. Sex-based differences in SRC incidence likely reflect a combination of physiological, biomechanical, and psychosocial factors. Physiologically, hormonal influences of estrogen and progesterone may affect symptom severity and recovery [18, 19]. Biomechanically, females tend to demonstrate lower head-neck strength and greater angular acceleration during impacts, increasing vulnerability [20–23]. Psychosocially, higher symptom awareness and greater willingness to report symptoms may also contribute to the higher observed SRC rates in female athletes [24].

The significant clinical, psychosocial, and economic impact of SRC highlights the importance of prevention as the most effective and ethically sound approach [25]. In this context, primary prevention refers to strategies implemented before an injury occurs, aiming to reduce the incidence of SRC through measures such as rule and policy modifications, protective equipment, targeted training interventions, and educational programs [1]. Primary prevention is particularly relevant because it aims to reduce the occurrence of concussion itself, whereas secondary and tertiary prevention primarily address injury recognition, clinical management, and long-term consequences after concussion has occurred [26]. It should be acknowledged that several widely implemented programs, such as Heads Up Football (HUF) [27] and RugbySmart [28], are hybrid in nature, combining primary prevention components with elements related to early recognition and post-injury management.

Considering the potential short- and long-term neurological consequences of SRC [29–31], primary prevention strategies warrant focused investigation. However, the evidence base informing current recommendations on these strategies remains limited. The 2022 Amsterdam Consensus Statement relied largely on a single systematic review and meta-analysis [32], and its recommendations for female athletes were based on only a small number of individual studies, resulting in insufficient evidence-based, sport-specific guidance for this population [32]. Following the publication of the Amsterdam statement, additional systematic reviews with or without meta-analyses have appeared, providing more detailed information but also presenting fragmented findings [25, 33–36]. This necessitates an updated and comprehensive synthesis of the available evidence to inform future SRC prevention efforts.

In this context, conducting an umbrella review (i.e., overview of systematic reviews) is appropriate for synthesizing and critically appraising the most current and comprehensive evidence on primary prevention strategies for SRC [37, 38]. Although a previous umbrella review reported SRC incidence rates [17], there is currently no comprehensive synthesis focused on evaluating the effectiveness of primary prevention strategies in reducing the incidence of SRC. Accordingly, we conducted an umbrella review to critically appraise and synthesize evidence from systematic reviews with or without metaanalyses on primary prevention strategies for SRC in athletes to identify effective strategies, clarify uncertainties, and guide future policy and practice.

## MATERIALS AND METHODS

The present umbrella review was conducted following the Cochrane Handbook for Overviews of Reviews [39] and reported in accordance with the Preferred Reporting Items for Overviews of Systematic Reviews Checklist (PRIOR) [40].

### Eligibility criteria

Studies were considered eligible based on the following inclusion criteria:

(a) Published or in-press peer-reviewed systematic reviews, with or without meta-analysis, written in any language. A study was classified as a systematic review if it collated evidence according to prespecified eligibility criteria to address a specific research question, using explicit and systematic methods to minimize bias [41];
(b) Evaluation of the effectiveness of any primary prevention strategy on the incidence of SRC. Because SRC is generally considered a subset of mild traumatic brain injury, studies referring to concussion within sport or exercise contexts were considered eligible. Hybrid programs were eligible only when they reported SRC incidence, allowing the extraction of the primary prevention effect; and
(c) Inclusion of competitive athletes of any age group or sex.

Systematic reviews of any study design were eligible, regardless of whether a meta-analysis was conducted. In contrast, systematic reviews that did not address the effectiveness of primary prevention strategies on SRC incidence or those focused solely on sedentary individuals or recreational athletes were excluded. Meta-analyses reporting results from mixed populations (e.g., recreational and competitive athletes) or mixed types of primary prevention strategies were also excluded if subgroup analyses were not provided.

To ensure the inclusion of studies representing the highest level of evidence, gray literature, narrative reviews, systematized reviews, and rapid reviews were excluded [42]. Additionally, original studies with primary data, editorials, letters, conference abstracts, posters, protocol registrations, and academic theses were not considered eligible.

### Search strategy

A comprehensive systematic literature search was conducted in eight databases, including the Cochrane Database of Systematic Reviews, Epistemonikos, ERIC, PsycINFO, PubMed, Scopus, SPORTDiscus, and Web of Science, up to 07 April 2025. The databases were selected to ensure broad coverage across biomedical, sport science, psychological, and educational research relevant to SRC prevention. A complementary search was conducted via Google Scholar on 10 April 2025 to identify any potentially missed studies [43,44]. However, due to the extensive number of results obtained, only the first 100 search records were screened [45]. An updated search was subsequently conducted in PubMed and Web of Science on 27 November 2025. The search used search terms and synonyms related to concussion (e.g., “concussion”, “mild traumatic brain injury”), sport (e.g., “athletes”, “sports”), prevention (e.g., “primary prevention,” “risk reduction”), and review type (e.g., “systematic review,” “meta-analysis”) combined with appropriate Boolean operators. Truncation, Medical Subject Headings (MeSH), and field tags were applied where relevant. In the search strategy, no language restriction was applied. However, only systematic reviews published from 2001 onward were considered. This cutoff was selected to enhance conceptual consistency, as studies published before the 2001 Consensus Statement on Concussion in Sport [46] may have relied on less standardized definitions of concussion. The 2001 statement provided the first widely recognized framework for defining concussion in athletic populations [46]. The reference lists of all included papers were manually screened to identify further relevant reviews. A detailed description of the searches with all keywords is presented in Supplemental Table S1.

### Study selection

The article selection process is illustrated in Figure 1. After removing duplicate records via EndNote X8, two authors (KT and HJ) independently screened titles and abstracts for eligibility. Discrepancies were resolved through discussion and mutual agreement. Full-text articles of potentially relevant studies were then retrieved and assessed by the same two authors, with disagreements resolved by consensus. The reasons for exclusion at the full-text stage are detailed in Supplemental Table S2. Additionally, the Google Scholar search was performed by the same two authors, with any discrepancies again resolved through mutual agreement.

**FIG. 1:**
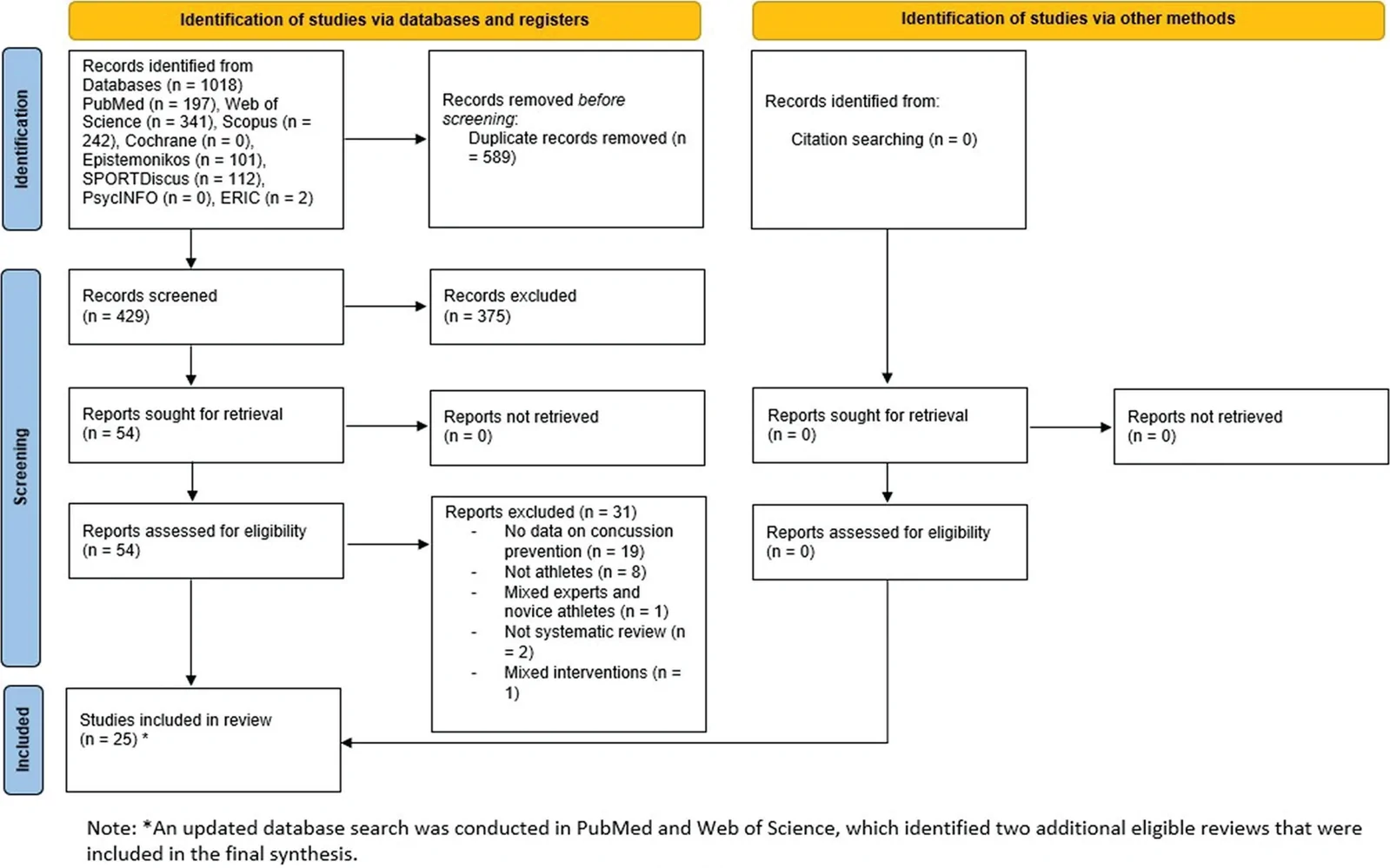
PRISMA flow diagram

### Data extraction

Two authors (KT and OB) independently extracted data from the included reviews via a standardized form developed and piloted specifically for our review. The extracted information included review characteristics (i.e., author, publication year, type of review, type of primary studies, databases searched, search timeframe, study designs, sample sizes, date range of included studies, quality assessment tools used), participant characteristics (i.e., sex, age range, country, sport practiced), and outcome-related data (i.e., number of included studies and corresponding sample sizes per outcome and/or exposure hours, effect sizes, certainty and/or level of evidence).

### Overlap of included studies

To assess the degree of overlap among primary studies across systematic reviews with meta-analyses, we calculated the corrected covered area (CCA) for each domain where more than one review was available [47]. The CCA quantifies the redundancy of primary studies by dividing the number of repeated occurrences of index studies (i.e., after their first inclusion) by the product of the number of index studies and the number of reviews minus the number of reviews [47]. The resulting value is expressed as a percentage ranging from 0% to 100%. The interpretation thresholds are as follows: 0%–5% indicates slight overlap, > 5%–10% moderate overlap, > 10%–15% high overlap, and > 15% very high overlap [47]. To visualize overlapping pairs of systematic reviews within each domain, a heatmap was generated via the ‘ccaR’ package in R (https://github.com/thdiakon/ccaR) [48, 49]. Further details are provided in the Online Supplemental Figure S1.

### Methodological quality assessment

The methodological quality of each included review was independently assessed by two reviewers (KT and HJ) via the AMSTAR-2 (A Measurement Tool to Assess Systematic Reviews) [50]. Any discrepancies were resolved through discussion until a consensus was reached. The AMSTAR-2 consists of 16 items, seven of which are considered critical (online supplemental Table S3) [50]. The AMSTAR-2 rates systematic reviews as critically low (more than one critical weakness, with or without noncritical weaknesses), low (one critical weakness, with or without noncritical weaknesses), moderate (no critical weakness, with more than one noncritical weakness), or high quality (neither critical nor noncritical weaknesses) [50].

### Data synthesis and analysis

Data reported in the included systematic reviews, with or without meta-analyses, were synthesized using the review as the unit of analysis. A narrative synthesis was conducted, with findings organized into four categories of intervention: (1) protective equipment, (2) rule or policy changes, (3) educational interventions, and (4) trainingbased approaches. Where available, pooled effect estimates (e.g., relative risks and incidence rate ratios [IRRs], with their 95% confidence intervals [CIs]) were extracted and presented in tabular format. Trends in effectiveness were examined across age groups, sport types, and implementation contexts. Given that systematic reviews with meta-analyses provide a higher level of evidence than those without quantitative synthesis [51], we assigned greater interpretative weight to findings from reviews that included meta-analyses when formulating our overall conclusions. Nevertheless, narrative results from systematic reviews without meta-analyses were also incorporated, particularly in areas where heterogeneity prevented pooling or where these reviews provided more detailed, sport-specific evidence.

### Interrater agreement

Interrater agreement was assessed at the screening and quality assessment stages via Cohen’s kappa coefficient, which adjusts for agreement occurring by chance [52]. Kappa values were interpreted according to the thresholds proposed by Landis and Koch [52], with values between 0.81 and 1.00 indicating almost perfect agreement.

### Protocol update

The review was conducted in accordance with the registered and publicly accessible protocol (Identifier: DOI 10.17605/OSF.IO/SQBDF). To ensure that the most recent studies were captured, an updated search of PubMed and Web of Science was conducted on 27 November 2025 in addition to the searches specified in the original protocol.

### Patient and public involvement

Patients and the public were not involved in the design, conduct, reporting, or dissemination of this umbrella review, because the study was based exclusively on previously published literature.

## RESULTS

### Study selection

The study selection process is presented in the PRISMA 2020 flow diagram (Figure 1). A total of 1,018 records were initially identified through electronic database searches. After removing duplicates, 429 unique references remained. Title and abstract screening resulted in the exclusion of 375 records, and 54 studies were subjected to full-text review. Of these, 31 did not meet the eligibility criteria and were excluded. Consequently, 23 reviews were included in the synthesis, to which two additional reviews identified through the updated PubMed and Web of Science search were added, resulting in a total of 25 included reviews. Interrater agreement between the two reviewers was high across all stages of the selection process, with Cohen’s kappa coefficients of 0.84 for title and abstract screening, 0.94 for full-text review, and 1.00 for the Google Scholar search.

### Study characteristics

Online supplemental Table S4 presents the characteristics of the 25 included systematic reviews with or without meta-analyses published between 2009 and 2025 [25, 32–36, 53–71]. Among these, eight systematic reviews incorporated meta-analyses on concussion primary prevention strategies [32–36, 53, 61, 65]. All reviews conducted searches in more than two major databases. Only two reviews included exclusively randomized controlled trials (RCTs) [33, 53]. Ice hockey, rugby, soccer, and American football were the most frequently studied sports, whereas only one review [70] focused on Olympic boxing. Most of the included reviews primarily focused on male athletes, whereas one review [35] focused specifically on female athletes.

### Methodological quality assessment

The methodological quality of the included reviews is presented in Table S5. All reviews were rated as critically low. None of the reviews justified their choice of study design for inclusion (Q3), and all failed to report the sources of funding for the primary studies (Q10). Furthermore, all the reviews only partially fulfilled the criterion for a comprehensive search strategy (Q4), which constitutes a critical methodological weakness. In addition, 20 out of 25 reviews did not provide a list of excluded studies with justifications (Q7), representing another critical methodological weakness. Interrater agreement between the two reviewers was high (mean Cohen’s kappa = 0.85).

### Overlap of included studies

Study overlap was high for mouthguards (CCA = 25%) and headgear reviews (CCA = 19.2%) and low for those on body checking (CCA = 0%).

### Policy, rule, or law changes

#### Results of SRs without meta-analyses

Rule and policy modifications demonstrated the strongest and most consistent evidence of effectiveness, particularly in contact and collision sports. Disallowing or delaying body checking in youth ice hockey, tackle football, and lacrosse consistently reduced SRC incidence across multiple reviews [32, 58, 62, 67]. Notably, the certainty of evidence for prohibiting body checking in youth ice hockey was high in the meta-analysis by Eliason et al. [32]. However, other policy changes, including zero-tolerance head contact rules, age-plus-weight player grouping, and tackling height reductions, have yielded limited or inconsistent results [32, 67, 69]. In rugby, lowering tackle height improved the technique but did not consistently reduce SRCs [69]. In soccer, concussion substitution rules and elbow-to-head penalties do not clearly reduce the incidence of SRC, and modifications to goal kick rules may offer only indirect benefits [69]. Evidence for female athletes was insufficient to draw conclusions [35].

#### Results of SRs with meta-analyses

Emery et al. [61] reported no significant effect of disallowing body checking on the incidence of SRC in ice hockey. In contrast, Eliason et al. [32] found that restricted body checking was associated with a 58% reduction in SRC among youth ice hockey players, with comparable reductions in both adolescent and child groups. In the same review, policy changes in American football were associated with a 64% reduction in SRC incidence overall [32]. Subgroup analyses revealed significant associations with 71% lower SRC rates with partial equipment use, 81% with reduced training intensity, and 70% with restricted collision time [32]. However, no significant association was found for policies targeting reduced contact during practices [32]. Age-stratified analyses also revealed significant associations, with SRC reductions of 56% in adolescents, 64% in adults, and 70% in mixed-sex groups [32]. Eliason et al. [32] further reported that rule changes in adult American football were not associated with any change in SRC incidence.

### Protective equipment

#### Headgear

– **Results of SRs without meta-analyses.** The evidence is insufficient to support the effectiveness of headgear in preventing SRCs among rugby and soccer players [55], Olympic boxers [70], and youth involved in collision sports [66].
– **Results of SRs with meta-analyses.** Al Attar et al. [53] conducted a Level 1a systematic review and meta-analysis of RCTs and found no significant effect of headgear use on the incidence of SRC in soccer and rugby players. Similarly, a meta-analysis across multiple sports (i.e., soccer, rugby, lacrosse, Australian football) found no overall significant effect [32]. However, in the sport-specific subgroup analysis, headgear use was associated with a statistically significant 36% reduction in SRC rates among soccer players [32].
  Shill et al. [35] conducted a female-only meta-analysis and similarly reported that a very low certainty of evidence suggested that headgear use was associated with a 30% reduction in SRC incidence in adolescent female lacrosse and soccer players.

#### Helmets

– **Results of SRs without meta-analyses.** There is no conclusive evidence supporting the effectiveness of helmets in preventing SRCs. Eliason et al. [32] reported inconsistent and contradictory findings, with some studies suggesting potential benefits from specific helmet types or designs (e.g., increased padding over the zygoma/mandible area, hard shell helmets with full facial protection) in sports such as American football, lacrosse, and ice hockey. However, the heterogeneity of the results limits the ability to recommend helmet use as a reliable primary prevention strategy [32]. Similarly, Benson et al. [55] reported no protective effect. In contrast, Emery et al. [61], on the basis of a single study, concluded that helmet use may reduce the risk of SRC in skiing and snowboarding. Additionally, Enniss et a. [62] recommended the adoption of newer helmet technologies in adult American football players, although supporting evidence remains limited.

#### Mouthguards

– **Results of SRs without meta-analyses.** Mouthguards use did not consistently reduce the risk of SRC in youth tackle football [71] or in rugby union players [57]. Additionally, dentist-fitted or specialized mouthguards have not been shown to offer greater protection than off-the-shelf models [32]. However, Pankow et al. [67] reported that custom-made mouthguards were associated with greater odds of SRC than non-custom mouthguards.
– **Results of SRs with meta-analyses.** No significant effect of mouthguards use on the incidence of SRC was reported in two metaanalyses, including basketball, ice hockey, and rugby union [61, 65]. Similarly, another meta-analysis, rated as low certainty, reported a significant 26% overall reduction in SRC risk with mouthguard use [32]. Subgroup analyses revealed significant reductions in SRC risk in ice hockey (28%), whereas no significant effect was observed in rugby union; reductions were also significant among adults (38%) and mixed-age groups (24%) [32].

#### Face shields/face guards

– **Results of SRs without meta-analyses.** Findings on the use of face shields (full, half, or none) in ice hockey are mixed and inconclusive [32, 54].

#### Eyewear

– **Results of SRs without meta-analyses.** There is currently no evidence supporting the effectiveness of protective eyewear in reducing the incidence of SRC [32].
– **Results of SRs with meta-analyses.** In adolescent female lacrosse athletes, a very low certainty of evidence indicated that eyewear use was not associated with a reduced incidence of SRC [35].

#### Jugular vein compression collars

– **Results of SRs without meta-analyses.** There is not sufficient evidence to recommend the use of jugular vein compression collars to reduce the incidence of SRC [25, 32].

### Training strategies

– **Results of SRs without meta-analyses.** Eliason et al. [32] reported that vision training and core strength programs were associated with reduced SRC rates in youth and adolescent athletes. Regular neuromuscular training (NMT) warm-ups, especially in rugby players, were associated with a reduced incidence of SRC [32]. Similarly, West et al. [71] concluded that completing an NMT program three times per week significantly reduced the degree of SRC risk.
  For multi-component programs such as HUF, Pankow et al. [67] concluded that participation in HUF, particularly when combined with contact restriction policies, was associated with reduced SRC rates in youth football. Moreover, high school players in non-HUF leagues experienced significantly higher SRC rates than those in HUF-affiliated leagues [67].
  Findings regarding neck strengthening were mixed. Daly et al. [59], Chavarro-Nieto et al. [56] and Peek et al. [68] reported insufficient evidence to support neck-strengthening interventions for SRC prevention. In contrast, Elliott et al. [60] indicated that injury prevention programs incorporating neck exercises may help reduce head and neck injuries, including SRCs. Elliott, Heron [60] offered conditional support for neck strengthening in adult amateur athletes, suggesting a potential benefit despite limited supporting evidence.
– **Results of SRs with meta-analyses.** Al Attar et al. [53] reported that the FIFA 11+ Kids program had no effect on the incidence of SRC in child soccer players. Similarly, Ivanic et al. [34] reported that although high-level evidence shows resistance training increases isometric neck strength, this does not mean that the gains will translate into a reduction in SRC incidence.
  Chen et al. [36] reported a significant 34% reduction in SRC incidence associated with injury-prevention programs. Subgroup analyses indicated that the effectiveness of these programs did not differ significantly between physical and educational interventions [36].

### Educational interventions

– **Results of SRs without meta-analyses.** Cusimano et al. [57] reported that educational initiatives (i.e., ‘’RugbySmart’’) were associated with a significant reduction in the incidence of SRC among rugby players. Fraas and Burchiel [64], however, reported that there was no available evidence supporting the effectiveness of concussion education programs for coaches or referees in preventing SRC. Feiss et al. [63] provided preliminary evidence suggesting that concussion education for coaches, particularly inperson training, may be associated with reduced SRC. The authors also noted that participation in the HUF program, which includes educational components, was associated with lower SRC rates in youth football [63]. Most recently, Eliason et al. [32] reported that comprehensive coach education programs, when combined with contact restrictions during practices, were associated with reduced SRC rates in youth football. Furthermore, the inclusion of a player safety coach was linked to additional reductions in practice-related SRCs, although no association was observed for game-related SRC incidence [67].

## DISCUSSION

Our overview shows that rule and policy modifications, particularly those that reduce exposure to high-risk contact in youth ice hockey, provide the most consistent evidence for preventing SRC. In contrast, evidence for protective equipment, educational, and training-based strategies remains inconsistent. Because all included systematic reviews were rated as critically low in methodological quality, the strength and reliability of the synthesized conclusions are limited. Higher-quality systematic reviews, along with rigorous and sportspecific primary studies, are needed to better determine the true effectiveness of SRC prevention strategies.

### Rules and policy changes

Rule and policy changes demonstrated the most consistent evidence for preventing SRC in contact and collision sports, particularly in youth ice hockey. A meta-analysis with high certainty of evidence reported that disallowing or delaying body checking in youth ice hockey reduced SRC incidence by 58%, likely due to decreased exposure to high-risk collisions [32]. Again, the contrasting findings compared with those of Emery et al. [61] may be explained by differences in study selection, including the exclusion of low-methodological-quality studies and the larger number of studies included in the meta-analysis by Eliason et al. [32].

In American football, strategies targeting practice-related concussion risk were effective in reducing the incidence of SRC, particularly among adolescents [32]. Specifically, partial equipment use, reduced training intensity, and restricted collision time were identified as the most effective measures [32]. However, these interventions were less effective in adult players, possibly because of greater baseline exposure to contact, greater physical intensity of play, and/or reduced adaptability to modified training protocols in older age groups [32]. Nevertheless, all these findings should be interpreted cautiously, as they are based on a limited number of studies, considerable heterogeneity across interventions and study designs, and, in some cases, exploratory subgroup comparisons.

Other policies, such as zero-tolerance head contact rules, tackle height reductions in rugby, and concussion substitutions in soccer, have also been examined and show limited or inconsistent effectiveness [32, 67, 69]. These mixed results could be attributed to enforcement challenges, a lower frequency of targeted events, and/or a limited impact on actual contact dynamics.

### Protective equipment

Most reviews reported no significant association between headgear use and reduced SRC risk across sports [32, 53, 55, 57, 66, 70]. This is likely because headgear is designed primarily to protect against superficial injuries (e.g., lacerations or abrasions) [72, 73] rather than mitigating the rotational and linear accelerations responsible for concussive injuries [74, 75]. In rugby union, for example, the use of headgear may inadvertently promote risk-compensation behaviors, particularly among youth players, who perceive it as offering more protection than it does [76, 77]. This misperception may introduce confounding factors into the approach, potentially encouraging more aggressive play, including the use of the head as a tackling tool [76, 77]. Such behavior modifications may indeed negate some or all of the potential concussion-preventing benefits of headgear and could even result in an increase in the risk of SRC [53, 76]. In contrast, in soccer, where SRCs more commonly result from aerial collisions, head-to-head contact, ball heading, or falls during tackles, meta-analytic findings have demonstrated a significant 36% reduction in SRC risk associated with headgear use in this sport [32]. This suggests that in sports involving lower-magnitude impacts and a lower likelihood of behavioral compensation, soft headgear may offer a degree of protection against SRC. Nevertheless, despite these encouraging findings, Eliason et al. [32] did not recommend headgear use in soccer, possibly due to the very low certainty and limited robustness of the available evidence. These observations were further supported by a Level 1a systematic review and meta-analysis of RCTs, which similarly reported no significant preventive effect [53].

Among female athletes, a meta-analysis in lacrosse and soccer reported a 30% reduction in SRC with headgear use [35]. However, the pooled analysis combined hard-shell and soft-shell headgear, which differ in design and biomechanical properties, limiting interpretability. The certainty of evidence was very low, the number of included studies was small, and heterogeneity was substantial. Consequently, the authors noted that these findings are likely to change with additional high-quality, sex-specific research and should not inform definitive recommendations [35]. This uncertainty is particularly important when considering female athletes, as sex-related biomechanical, physiological, and psychosocial factors may influence SRC risk and injury reporting patterns [18–24]. Overall, these limitations highlight the need for further studies that accounts for headgear design, sport-specific demands, sex-related differences in SRC risk, and variability in concussion definitions.

For helmets, a narrative synthesis reported no consistent preventive effect across sports [32]. Although helmets reduce the risk of skull fractures, they are less effective in mitigating brain movement within the skull, especially in high-speed collisions.

Some reviews have suggested potential benefits of newer helmet technologies in specific contexts, such as skiing and snowboarding, based on one individual study [61] and adult American football [62]; however, these findings are not generalizable across sports. Additionally, Pankow et al. [67] reported differences in SRC rates between helmet models and brands, with helmets featuring thicker zygoma pads associated with reduced SRC risk. Furthermore, poor helmet fit was linked to more severe and prolonged concussion symptoms, whereas helmet age (i.e., time since initial use) and reconditioning were not associated with SRC outcomes [67].

These mixed findings likely reflect heterogeneity in helmet technology, sport-specific impact profiles, fit quality, and study methodology rather than true differences in efficacy. Eliason et al. [32] emphasized the need for future research on helmet fit and padding characteristics, highlighting important gaps in current evidence regarding their effectiveness as primary prevention strategies for SRCs.

Mouthguards were originally designed to prevent orofacial injuries [78], and their role in SRC prevention remains debated. Previous reviews [57, 61, 71] did not find consistent evidence supporting a protective effect against SRC, likely due to methodological heterogeneity, low adherence among athletes, and/or variability in mouthguard types, particularly between custom-fit and stock models. Similarly, a meta-analysis by Emery et al. [61] failed to demonstrate their significant preventive effect.

A more recent systematic review and meta-analysis, which excluded low-methodological-quality studies, reported a 26% overall reduction in SRC risk associated with mouthguards use [32]; however, because the review itself was rated as critically low in quality and the certainty of evidence was low, these findings should be interpreted with caution. The subgroup reductions of 28% in ice hockey and 38% in adult athletes were derived from observational data within the meta-analysis [32], further limiting causal inference. Although these results may suggest a potential protective effect, possibly through improved jaw positioning and attenuation of impact forces transmitted to the skull [65, 79, 80], the underlying evidence base remains weak, and firm conclusions cannot be drawn. It should be acknowledged that dentist-fitted or specialized mouthguards have not demonstrated superior protective effects compared with off-the-shelf models [32], possibly because SRC prevention relies more on general impact attenuation than on the degree of customization, with both types providing similar cushioning of the jaw. Moreover, Pankow et al. [67] observed higher odds of SRC among users of custom-made mouthguards compared with standard models, a finding the authors speculated may relate to increased risk-taking or overconfidence in the perceived protection.

Despite these mixed findings, continued innovations in mouthguards design and further high-quality research are warranted to clarify their preventive value across different sports and age groups [81].

The effectiveness of face shields or face guards in preventing SRC remains unclear. Available systematic reviews reported mixed and inconclusive findings regarding the use of full, half, or no face shields in ice hockey [32, 54]. While face shields are effective in preventing facial injuries (e.g., lacerations and fractures), their role in reducing concussive impacts is less evident. This is likely because face guards primarily protect the face rather than influencing the intracranial rotational or linear forces that contribute to SRC. Further highquality studies are needed to determine whether specific shield designs or combinations with other equipment offer any measurable protection against SRCs.

Eyewear demonstrated no effect on the incidence of SRC in male and adolescent female athletes [32, 35], which is expected, given that its role is limited to eye protection and does not influence the biomechanical forces involved in concussion.

Jugular vein compression collars have been proposed to stabilize the brain by increasing the intracranial blood volume [25]. However, the included reviews [25, 32] found insufficient evidence to support their use. It is worth noting that most studies were preliminary or underpowered, and no studies have yet confirmed consistent efficacy in real-world settings.

### Educational interventions

Educational interventions show promise in reducing SRC, particularly when targeting coaches and when implemented alongside structural measures such as practice contact restrictions. Programs such as ‘’RugbySmart’’ are associated with a lower incidence of concussion among rugby union players [57]. These effects are likely due to improved coaching decisions (e.g., modifying drills to reduce contact and managing practice intensity to minimize head impact exposure). Similarly, the HUF program, which combines education with technique instruction and practice modifications, has been associated with a reduced incidence of concussion in youth football [67]. However, these benefits are less consistent during games, where coaches have less control over contact dynamics, and players definitely take more risks compared to training [32]. The addition of roles such as player safety coaches further enhanced practice-related outcomes [32, 67], emphasizing the value of real-time supervision. Overall, concussion education appears most effective when it is integrated into broader, sport-specific strategies rather than being delivered as isolated or passive efforts [63, 64].

### Training-based interventions

When weighted according to the strength of evidence, systematic reviews with meta-analyses provide limited and inconclusive support for training-based interventions as standalone strategies for preventing SRC. Neither the FIFA 11+ Kids program [53] nor the NMT warm-ups [34] demonstrated significant reductions in SRC incidence. Although conceptually plausible and designed to enhance balance, coordination, neuromuscular control, and preparedness for contact, these interventions lack consistent empirical support, possibly because of small sample sizes, inconsistent implementation, and variable adherence. The only meta-analysis reporting a significant reduction in SRC incidence was that of Chen et al. [36], who found a 34% decrease in SRC risk across broad injury-prevention programs. However, subgroup analysis revealed no significant difference between physical and educational components [36], indicating that the observed protective effect cannot be attributed solely to training-based interventions. Collectively, the meta-analytic evidence suggests no reliable benefit of training strategies when isolated from broader, multi-component approaches.

Findings from systematic reviews without meta-analysis, though informative, serve mainly as contextual and hypothesis-generating evidence. These narrative reviews suggest that vision training, core strengthening, NMT warm-ups in rugby players, and multi-component programs such as HUF may be associated with reduced SRC rates [32, 67, 71]. However, the absence of pooled estimates and the reliance on heterogeneous study designs limit their contribution to firm conclusions, and they cannot outweigh the null meta-analytic evidence.

Similarly, evidence on neck strengthening remains inconsistent. Some reviews conclude that current data are insufficient to support neck-strengthening programs as preventive strategies [56, 59, 68], while others report potential benefits when neck exercises are embedded within broader injury-prevention frameworks [60, 62]. Again, without meta-analytic confirmation and given substantial variability across studies, confidence in neck strengthening as an effective, standalone SRC-prevention strategy remains low.

### Strengths and limitations

This overview has several strengths, including adherence to Cochrane and PRISMA guidelines, a comprehensive multi-database search, AMSTAR-2 appraisal, and assessment of study overlap using CCA. However, several limitations must be acknowledged. All included reviews were rated as critically low quality according to AMSTAR-2, reflecting important methodological limitations that reduce confidence in the reliability and transparency of the synthesized evidence. In particular, weaknesses related to search comprehensiveness, reporting transparency, and justification of methodological choices suggest that potentially relevant studies may have been missed and that the reproducibility of some reviews may be limited. Consequently, the conclusions of the existing evidence syntheses should be interpreted with caution. The certainty of evidence was also not assessed using frameworks such as Grading of Recommendations, Assessment, Development and Evaluation (GRADE) [82], and most primary studies were observational, limiting causal inference. Additionally, although data extraction was conducted independently by two reviewers, inter-rater agreement was not calculated for this stage and should be incorporated in future reviews to enhance transparency and reproducibility. Substantial overlap was observed for certain intervention domains (i.e., headgear, mouthguards), indicating that several systematic reviews relied on many of the same primary studies. This redundancy reduces the independence of the evidence base and may inflate the apparent consistency or strength of the synthesized findings. Consequently, conclusions regarding the effectiveness of headgear and mouthguards should be interpreted with caution. Furthermore, the high degree of overlap precluded the conduct of a meta-analysis of meta-analyses. Together, these limitations highlight the need for higher-quality primary research rather than additional low-quality systematic reviews.

### Research agenda

First, future research should prioritize well-powered and methodologically rigorous RCTs to evaluate the effectiveness of helmet, headgear, and/or mouthguard designs, particularly in sports such as soccer, where headgear shows promising results. Adequate sample sizes determined through a priori statistical power calculations [83, 84] will be essential to ensure sufficient ability to detect meaningful changes in SRC incidence. Second, although placebo or sham groups have been proposed to better determine the true effect of protective equipment [85], such designs raise ethical concerns because they may require withholding established protective equipment. Consequently, researchers should explore alternative approaches, such as comparative-effectiveness designs, biomechanical evaluations, or blinding of outcome assessors, to advance the evidence base without compromising athlete safety. Third, studies should determine how rule changes, training programs, and educational strategies can be optimized across different sports, ages, and competition levels. Fourth, training-focused research is needed to determine the ideal frequency and sport-specific tailoring of neuromuscular, vision, and neckstrengthening exercises. Fifth, greater attention must be directed toward underrepresented groups, including female athletes, paraathletes, and combat sport participants. In particular, future studies should include sex-stratified analyses when possible to better understand potential sex-specific differences in SRC risk and the effectiveness of prevention strategies. Last, multicomponent interventions that integrate education, equipment, rule enforcement, and training warrant evaluation, and future work should incorporate implementation science frameworks such as RE-AIM to evaluate Reach, Effectiveness, Adoption, Implementation, and Maintenance in applied sport settings [86, 87].

### Clinical implications

Primary prevention of SRC should prioritize strategies supported by the most consistent evidence, particularly rule and policy changes that reduce exposure to high-risk contact (i.e., restricting body checking in youth ice hockey). Educational initiatives may also contribute to risk reduction, but only when combined with concrete structural modifications rather than used in isolation. Equipment such as helmets, headgear, and mouthguards provides important protection against other injuries but shows uncertain and inconsistent signals of benefit for SRC prevention. Training programs, including NMT warm-ups and neck strengthening, may offer complementary advantages but currently lack strong or consistent evidence to support their use as stand-alone strategies.

## CONCLUSIONS

This overview indicates that rule and policy modifications, especially those reducing exposure to high-risk contact, provide the most consistent evidence for preventing SRC. In contrast, evidence supporting protective equipment, educational, and training-based strategies remains mixed and inconsistent. Because all included systematic reviews were assessed as critically low in methodological quality, confidence in the synthesized conclusions is limited. More robust systematic reviews and well-designed, sport-specific primary studies are required to determine the true effectiveness of SRC prevention strategies.

## Supplementary Material

What evidence supports primary prevention strategies for sport-related concussion? An umbrella review

## Data Availability

Data sharing not applicable as no datasets generated and/or analysed for this study.
