# Supplementary material for: What evidence supports primary prevention strategies for sport-related concussion? An umbrella review
Source: Biol Sport. 2026 Jul 13;43:1589–614. doi: 10.5114/biolsport.2026.161108 (PMC13711644; doi:10.5114/biolsport.2026.161108)
Supplement: Supplementary file 1 [file JBS-43-57926-s1.pdf]

SUPPLEMENTARY MATERIALS

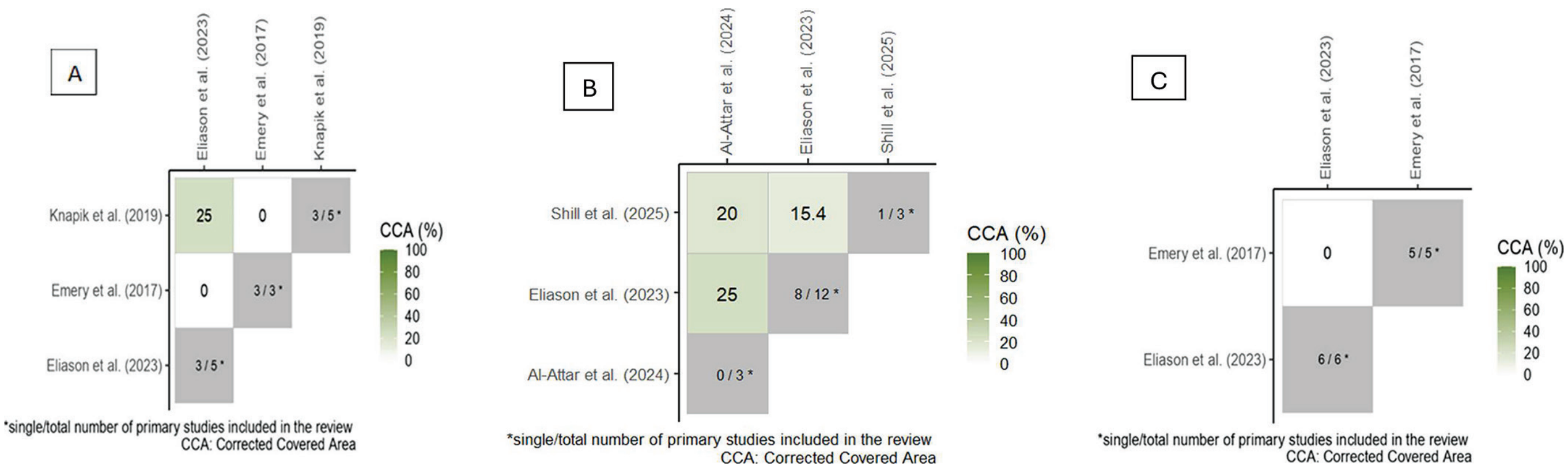

FIG. S1. Overlap of included studies on mouthguards (A), headgear (B), and body checking (C).

TABLE S1. Search strategy.

**PubMed (07-05-2025)**		
**[1]:** (concussion* [tw] OR "brain concussion" [tw] OR mild traumatic brain injur* [tw] OR "mild TBI" [tw] OR mTBI [tw] OR "sports-related concussion" [tw] OR "Head Injuries, Closed" [Mesh] OR head injur* [tw] OR "craniocerebral trauma" [tw] OR "Craniocerebral Trauma" [Mesh] OR craniocerebral injur* [tw] OR neurotrauma* [tw] OR neuro-trauma [tw])	124,895	[1] AND [2] AND [3] AND [4] = 197
**[2]:** (prevent* [tw] OR "Primary Prevention" [MeSH] OR prophylaxis [tw] OR "risk reduction" [tw] OR mitigation [tw] OR "Head Protective Devices" [Mesh] OR head protective device* [tw] OR helmet* [tw] OR "Sports Equipment" [Mesh] OR sports equipment* [tw] OR head protect* [tw] OR neck protect* [tw] OR mouth protect* [tw] OR face protect* [tw] OR headgear* [tw] OR head gear* [tw] OR mask* [tw] OR collar* [tw] OR q-collar* [tw] OR visor* [tw] OR goggle* [tw] OR mouthguard* [tw] OR mouth guard* [tw] OR mouthpiece* [tw] OR mouth piece* [tw] OR woodpecker* [tw] OR spotter* [tw] OR "jugular vein compression" [tw] OR law [tw] OR "Jurisprudence" [Mesh] OR jurisprudence [tw] OR "Legislation as Topic" [Mesh] OR legislation [tw] OR rule* [tw] OR policy [tw] OR policies [tw] OR regulation* [tw] OR "Muscle Strength" [Mesh] OR "muscle strength" [tw] OR "Resistance Training" [Mesh] OR "resistance training" [tw] OR "Proprioception" [Mesh] OR proprioception [tw] OR neuromuscular [tw] OR "Education" [Mesh] OR education [tw] OR "Dietary Supplements" [Mesh] OR dietary supplement* [tw] OR nutritional supplement* [tw])	5,586,811	
**[3]:** ("athletes" [MeSH] OE athlet* [tw] OR "Sports" [MeSH] OR sport* [tw] OR "contact sports" [tw] OR "Youth Sports" [Mesh] OR "youth sports" [tw] OR "professional athletes" [tw] OR "Team Sports" [Mesh] OR "team sports" [tw])	248,600	
**[4]:** ("Systematic Review" [Publication Type] OR "systematic review" [tiab] OR "systematic literature review" [tiab] OR "Meta-Analysis" [Publication Type] OR "systematic review of the literature" [tiab] OR meta-analysis [tiab] OR "meta-analytical review" [tiab] OR "systematic review and meta-analysis" [tiab] OR "systematic review with meta-analysis" [tiab])	517,697	
**Web of Science (07-05-2025)**		
**[1]:** (concussion* OR "brain concussion" OR mild traumatic brain injur* OR "mild TBI" OR mTBI OR "sports-related concussion" OR head injur* OR "craniocerebral trauma" OR craniocerebral injur* OR neurotrauma* OR neuro-trauma)	112,257	[1] AND [2] AND [3] AND [4] = 341
**[2]:** (prevent* OR "primary prevention" OR prophylaxis OR "risk reduction" OR mitigation OR head protective device* OR helmet* OR sports equipment* OR head protect* OR neck protect* OR mouth protect* OR face protect* OR headgear* OR head gear* OR mask* OR collar* OR q-collar* OR visor* OR goggle* OR mouthguard* OR mouth guard* OR mouthpiece* OR mouth piece* OR woodpecker* OR spotter* OR "jugular vein compression" OR law OR jurisprudence OR legislation OR rule* OR policy OR policies OR regulation* OR "muscle strength" OR "resistance training" OR proprioception OR neuromuscular OR education OR dietary supplement* OR nutritional supplement*)	14,202,201	
**[3]:** (athlet* OR sport* OR "contact sports" OR "youth sports" OR "professional athletes" OR "team sports")	1,558,178	
**[4]:** ("systematic review" OR "systematic literature review" OR meta-analysis OR "systematic review of the literature" OR "meta-analytical review" OR "systematic review and meta-analysis" OR "systematic review with meta-analysis")	644,239	

TABLE S1. Continue

**Scopus (07-05-2025)**		
**[1]:** (concussion* OR “brain concussion” OR “mild traumatic brain injur*” OR “mild TBI” OR mTBI OR “sports-related concussion” OR “head injur*” OR “craniocerebral trauma” OR “craniocerebral injur*” OR neurotrauma* OR neuro-trauma)	67,729	**[1] AND [2] AND [3] AND [4]** = 242
**[2]:** (prevent* OR “primary prevention” OR prophylaxis OR “risk reduction” OR mitigation OR “head protective device*” OR helmet* OR “sports equipment*” OR “head protect*” OR “neck protect*” OR “mouth protect*” OR “face protect*” OR headgear* OR “head gear*” OR mask* OR collar* OR q-collar* OR visor* OR goggle* OR mouthguard* OR “mouth guard*” OR mouthpiece* OR “mouth piece*” OR woodpecker* OR spotter* OR “jugular vein compression” OR law OR jurisprudence OR legislation OR rule* OR policy OR policies OR regulation* OR “muscle strength” OR “resistance training” OR proprioception OR neuromuscular OR education OR “dietary supplement*” OR “nutritional supplement*”)	11,496,266	
**[3]:** (athlet* OR sport* OR “contact sports” OR “youth sports” OR “professional athletes” OR “team sports”)	402,389	
**[4]:** (“systematic review” OR “systematic literature review” OR meta-analysis OR “systematic review of the literature” OR “meta-analytical review” OR “systematic review and meta-analysis” OR “systematic review with meta-analysis”)	840,014	
**Cochrane Database of Systematic Reviews (07-05-2025)**		
**[1]:** (concussion* OR “brain concussion” OR mild traumatic brain injur* OR “mild TBI” OR mTBI OR “sports-related concussion” OR head injur* OR “craniocerebral trauma” OR craniocerebral injur* OR neurotrauma* OR neuro-trauma)	93	**[1] AND [2] AND [3] AND [4]** = 0
**[2]:** (prevent* OR “primary prevention” OR prophylaxis OR “risk reduction” OR mitigation OR head protective device* OR helmet* OR sports equipment* OR head protect* OR neck protect* OR mouth protect* OR face protect* OR headgear* OR head gear* OR mask* OR collar* OR q-collar* OR visor* OR goggle* OR mouthguard* OR mouth guard* OR mouthpiece* OR mouth piece* OR woodpecker* OR spotter* OR “jugular vein compression” OR law OR jurisprudence OR legislation OR rule* OR policy OR policies OR regulation* OR “muscle strength” OR “resistance training” OR proprioception OR neuromuscular OR education OR dietary supplement* OR nutritional supplement*)	4951	
**[3]:** (athlet* OR sport* OR “contact sports” OR “youth sports” OR “professional athletes” OR “team sports”)	136	
**[4]:** (“systematic review” OR “systematic literature review” OR meta-analysis OR “systematic review of the literature” OR “meta-analytical review” OR “systematic review and meta-analysis” OR “systematic review with meta-analysis”))	4497	
**Epistemonikos (07-05-2025)**		
**[1]:** (concussion* OR “brain concussion” OR “mild traumatic brain injur*” OR “mild TBI” OR mTBI OR “sports-related concussion” OR “head injur*” OR “craniocerebral trauma” OR “craniocerebral injur*” OR neurotrauma* OR neuro-trauma)	5,391	**[1] AND [2] AND [3] AND [4]** = 101
**[2]:** (prevent* OR “primary prevention” OR prophylaxis OR “risk reduction” OR mitigation OR “head protective device*” OR helmet* OR “sports equipment*” OR “head protect*” OR “neck protect*” OR “mouth protect*” OR “face protect*” OR headgear* OR “head gear*” OR mask* OR collar* OR q-collar* OR visor* OR goggle* OR mouthguard* OR “mouth guard*” OR mouthpiece* OR “mouth piece*” OR woodpecker* OR spotter* OR “jugular vein compression” OR law OR jurisprudence OR legislation OR rule* OR policy OR policies OR regulation* OR “muscle strength” OR “resistance training” OR proprioception OR neuromuscular OR education OR “dietary supplement*” OR “nutritional supplement*”)	1,216,377	
**[3]:** (athlet* OR sport* OR “contact sports” OR “youth sports” OR “professional athletes” OR “team sports”)	67,785	
**[4]:** (“systematic review” OR “systematic literature review” OR meta-analysis OR “systematic review of the literature” OR “meta-analytical review” OR “systematic review and meta-analysis” OR “systematic review with meta-analysis”)	523,967	

TABLE S1. Continue

**SPORTDiscus (07-05-2025)**			
**[1]:**	(concussion* OR “brain concussion” OR “mild traumatic brain injur*” OR “mild TBI” OR mTBI OR “sports-related concussion” OR “head injur*” OR “craniocerebral trauma” OR “craniocerebral injur*” OR neurotrauma* OR neuro-trauma)	9,086	**[1] AND [2] AND [3] AND [4]** = 112
**[2]:**	(prevent* OR “primary prevention” OR prophylaxis OR “risk reduction” OR mitigation OR “head protective device*” OR helmet* OR “sports equipment*” OR “head protect*” OR “neck protect*” OR “mouth protect*” OR “face protect*” OR headgear* OR “head gear*” OR mask* OR collar* OR q-collar* OR visor* OR goggle* OR mouthguard* OR “mouth guard*” OR mouthpiece* OR “mouth piece*” OR woodpecker* OR spotter* OR “jugular vein compression” OR law OR jurisprudence OR legislation OR rule* OR policy OR policies OR regulation* OR “muscle strength” OR “resistance training” OR proprioception OR neuromuscular OR education OR “dietary supplement*” OR “nutritional supplement*”)	434,530	
**[3]:**	(athlet* OR sport* OR “contact sports” OR “youth sports” OR “professional athletes” OR “team sports”)	858,327	
**[4]:**	(“systematic review” OR “systematic literature review” OR meta-analysis OR “systematic review of the literature” OR “meta-analytical review” OR “systematic review and meta-analysis” OR “systematic review with meta-analysis”)	23,782	
**PsycINFO (07-05-2025)**			
**[1]:**	(concussion* OR “brain concussion” OR “mild traumatic brain injur*” OR “mild TBI” OR mTBI OR “sports-related concussion” OR “head injur*” OR “craniocerebral trauma” OR “craniocerebral injur*” OR neurotrauma* OR neuro-trauma)	17107	**[1] AND [2] AND [3] AND [4]** = 23
**[2]:**	(prevent* OR “primary prevention” OR prophylaxis OR “risk reduction” OR mitigation OR “head protective device*” OR helmet* OR “sports equipment*” OR “head protect*” OR “neck protect*” OR “mouth protect*” OR “face protect*” OR headgear* OR “head gear*” OR mask* OR collar* OR q-collar* OR visor* OR goggle* OR mouthguard* OR “mouth guard*” OR mouthpiece* OR “mouth piece*” OR woodpecker* OR spotter* OR “jugular vein compression” OR law OR jurisprudence OR legislation OR rule* OR policy OR policies OR regulation* OR “muscle strength” OR “resistance training” OR proprioception OR neuromuscular OR education OR “dietary supplement*” OR “nutritional supplement*”)	1350124	
**[3]:**	(athlet* OR sport* OR “contact sports” OR “youth sports” OR “professional athletes” OR “team sports”)	66612	
**[4]:**	(“systematic review” OR “systematic literature review” OR meta-analysis OR “systematic review of the literature” OR “meta-analytical review” OR “systematic review and meta-analysis” OR “systematic review with meta-analysis”)	92507	
**ERIC (07-05-2025)**			
**[1]:**	(concussion* OR “brain concussion” OR “mild traumatic brain injur*” OR “mild TBI” OR mTBI OR “sports-related concussion” OR “head injur*” OR “craniocerebral trauma” OR “craniocerebral injur*” OR neurotrauma* OR neuro-trauma)	763	**[1] AND [2] AND [3] AND [4]** = 2
**[2]:**	(prevent* OR “primary prevention” OR prophylaxis OR “risk reduction” OR mitigation OR “head protective device*” OR helmet* OR “sports equipment*” OR “head protect*” OR “neck protect*” OR “mouth protect*” OR “face protect*” OR headgear* OR “head gear*” OR mask* OR collar* OR q-collar* OR visor* OR goggle* OR mouthguard* OR “mouth guard*” OR mouthpiece* OR “mouth piece*” OR woodpecker* OR spotter* OR “jugular vein compression” OR law OR jurisprudence OR legislation OR rule* OR policy OR policies OR regulation* OR “muscle strength” OR “resistance training” OR proprioception OR neuromuscular OR education OR “dietary supplement*” OR “nutritional supplement*”)	836,401	
**[3]:**	(athlet* OR sport* OR “contact sports” OR “youth sports” OR “professional athletes” OR “team sports”)	16,224	
**[4]:**	(“systematic review” OR “systematic literature review” OR meta-analysis OR “systematic review of the literature” OR “meta-analytical review” OR “systematic review and meta-analysis” OR “systematic review with meta-analysis”)	13,223	

Updated search

Web of Science (27-11-2025)

[1] AND [2] AND [3] AND [4] = 30

PubMed (27-11-2025)

[1] AND [2] AND [3] AND [4] = 24

TABLE S2. List of excluded studies with reasons.

Studies	Exclusion reasons
1. Baker R, Bond B, Irwin G, et al. (2025). A systematic review of concussion education, knowledge, and attitudes in football. Science and Medicine in Football. https://doi.org/10.1080/24733938.2025.2488521	No data on concussion incidence prevention
2. Bigdon SF, Hecht V, Fairhurst PG, et al. (2022). Injuries in alpine summer sports—types, frequency and prevention: A systematic review. BMC Sports Science, Medicine and Rehabilitation, 14(1). https://doi.org/10.1186/s13102-022-00468-4	No data on concussion incidence prevention
3. Büth CM, Barbour N, Abdel-Aty M. (2023). Effectiveness of bicycle helmets and injury prevention: A systematic review of meta-analyses. Scientific Reports, 13(1), 8540. https://doi.org/10.1038/s41598-023-35728-x	Not athletes
4. Cigrovski V. (2018). Ski helmets: Are they preventing injuries of recreational alpine skiers?—An evidence-based review. Croatian Sports Medicine Journal, 33(2), 65–71.	Not athletes
5. Conaghan C, Daly E, Pearce AJ, et al. (2021). A systematic review of the effects of educational interventions on knowledge and attitudes towards concussion for people involved in sport—Optimising concussion education based on current literature. Journal of Sports Sciences, 39(5), 552–567. https://doi.org/10.1080/02640414.2020.1835223	No data on concussion incidence prevention
6. Cornelissen M, Kemler E, Verhagen E, et al. (2020). A systematic review of injuries in recreational field hockey: From injury problem to prevention. Journal of Sports Sciences, 38(17), 1953–1974. https://doi.org/10.1080/02640414.2020.1764898	No data on concussion incidence prevention
7. Cusimano MD, Kwok J. (2010). The effectiveness of helmet wear in skiers and snowboarders: A systematic review. British Journal of Sports Medicine, 44(11), 781–786. https://doi.org/10.1136/bjsm.2009.070573	No data on concussion incidence prevention
8. Cusimano MD, Zhu A. (2017). Systematic review of traumatic brain injuries in baseball and softball: A framework for prevention. Frontiers in Neurology, 8. https://doi.org/10.3389/fneur.2017.00492	No data on concussion incidence prevention
9. Ding L, Luo J, Smith DM, et al. (2022). Effectiveness of warm-up intervention programs to prevent sports injuries among children and adolescents: A systematic review and meta-analysis. International Journal of Environmental Research and Public Health, 19(10). https://doi.org/10.3390/ijerph19106336	No data on concussion incidence prevention
10. Fan Z, Min L, H, W, et al. (2024). Efficacy of multicomponent interventions on injury risk among ice and snow sports participants—A systematic review and meta-analysis. BMC Sports Science, Medicine and Rehabilitation, 16(1). https://doi.org/10.1186/s13102-024-00921-6	No data on concussion incidence prevention
11. Faude O, Rößler R, Junge A. (2013). Football injuries in children and adolescent players: Are there clues for prevention? Sports Medicine, 43(9), 819–837. https://doi.org/10.1007/s40279-013-0061-x	No data on concussion incidence prevention
12. Fitzpatrick DG, Goh M, Howlett DC, et al. (2018). Bicycle helmets are protective against facial injuries, including facial fractures: A meta-analysis. International Journal of Oral and Maxillofacial Surgery, 47(9), 1121–1125. https://doi.org/10.1016/j.ijom.2018.03.005	Not athletes
13. Hébert-Losier K, Holmberg HC. (2013). What are the exercise-based injury prevention recommendations for recreational alpine skiing and snowboarding? A systematic review. Sports Medicine, 43(5), 355–366. https://doi.org/10.1007/s40279-013-0032-2	Not athletes
14. Hussain A, Malcolm D, Tausif M. (2024). A systematic review of sports-related concussion education programs: Design, outcomes, and recommendations. Clinical Journal of Sport Medicine. Advance online publication. https://doi.org/10.1097/JSM.0000000000001309	No data on concussion incidence prevention
15. Indharty RS, Siahaan AMP, Susanto M, et al. (2023). Prevention of sports-related concussion in soccer: A comprehensive review of the literature. Annals of Medicine and Surgery, 85(3), 365–373. https://doi.org/10.1097/MS9.0000000000000268	Not a systematic review
16. Karkhaneh M, Kalenga IC, Hagel BE, et al. (2006). Effectiveness of bicycle helmet legislation to increase helmet use: A systematic review. Injury Prevention, 12(2), 76–82. https://doi.org/10.1136/ip.2005.010942	Not athletes

TABLE S2. Continue

Studies	Exclusion reasons
17. Knapik JJ, Hoedebecke BL, Mitchener TA. (2020). Mouthguards for the prevention of orofacial injuries in military and sports activities: Part 2, effectiveness of mouthguard for protection from orofacial injuries. Journal of Special Operations Medicine, 20(3), 114–116. https://doi.org/10.55460/ifcd-6d3a	No data on concussion incidence prevention
18. Macpherson A, Spinks A. (2008). Bicycle helmet legislation for the uptake of helmet use and prevention of head injuries. Cochrane Database of Systematic Reviews, 2008(3), CD005401. https://doi.org/10.1002/14651858.CD005401.pub3	Not athletes
19. McBain K, Shrier I, Shultz R, et al. (2012). Prevention of sport injury II: A systematic review of clinical science research. British Journal of Sports Medicine, 46(3), 174–179. https://doi.org/10.1136/bjsm.2010.081182	No data on concussion incidence prevention
20. McCradden MD, Cusimano MD. (2018). Concussions in sledding sports and the unrecognized “sled head”: A systematic review. Frontiers in Neurology, 9. https://doi.org/10.3389/fneur.2018.00772	No data on concussion incidence prevention
21. McGuine T. (2006). Sports injuries in high school athletes: A review of injury-risk and injury-prevention research. Clinical Journal of Sport Medicine, 16(6), 488–499. https://doi.org/10.1097/01.jsm.0000248848.62368.43	No data on concussion incidence prevention
22. Michael DC. (2010). The effectiveness of helmet wear in skiers and snowboarders: A systematic review. British Journal of Sports Medicine, 44(11), 781–786. https://doi.org/10.1136/bjsm.2009.070573	No data on concussion incidence prevention
23. Olivier J, Creighton P. (2017). Bicycle injuries and helmet use: A systematic review and meta-analysis. International Journal of Epidemiology, 46(1), 278–292. https://doi.org/10.1093/ije/dyw153	Not athletes
24. Phillips N, Crisco JJ. (2020). The effectiveness of regulations and behavioral interventions on head impacts and concussions in youth, high-school, and collegiate football: A systematized review. Annals of Biomedical Engineering, 48(11), 2508–2530. https://doi.org/10.1007/s10439-020-02624-8	Systematized review
25. Pocecco E, Ruedl G, Stankovic N, et al. (2013). Injuries in judo: A systematic literature review including suggestions for prevention. British Journal of Sports Medicine, 47(18), 1139–1143. https://doi.org/10.1136/bjsports-2013-092886	No data related to concussion incidence prevention
26. Mrazik M, Dennison CR, Brooks BL, et al. (2009). Knowledge transfer principles as applied to sport concussion education. British Journal of Sports Medicine, 43(Suppl 1), i68–i75.	No data related to concussion incidence prevention
27. Ramírez L. (2024). Biomechanical strategies for injury prevention in adolescent athletes: A systematic review. International Journal of Medicine and Science of Physical Activity and Sport, 24(98), 142–156. https://doi.org/10.15366/rimcafd2024.98.010	No data related to concussion incidence prevention
28. Richards D. (2018). Bicycle helmet use and facial injuries. Evidence-Based Dentistry, 19(4), 113. https://doi.org/10.1038/sj.ebd.6401344	Not athletes
29. Robles-Palazón FJ, Blázquez-Rincón D, López-Valenciano A, et al. (2024). A systematic review and network meta-analysis on the effectiveness of exercise-based interventions for reducing the injury incidence in youth team-sport players. Part 1: An analysis by classical training components. Annals of Medicine, 56(1). https://doi.org/10.1080/07853890.2024.2408457	No data related to concussion incidence prevention
30. Russell K, Christie J, Hagel BE. (2010). The effect of helmets on the risk of head and neck injuries among skiers and snowboarders: A meta-analysis. CMAJ Canadian Medical Association Journal, 182(4), 333–340. https://doi.org/10.1503/cmaj.091080	Mixed experts and novice athletes
31. Schneider DK, Grandhi RK, Bansal P, et al. (2017). Current state of concussion prevention strategies: A systematic review and meta-analysis of prospective, controlled studies. British Journal of Sports Medicine, 51(20), 1473–1482. https://doi.org/10.1136/bjsports-2015-095645	Mixed interventions

TABLE S3. Methodological quality of included studies.

Review	Q1	Q2[#]	Q3	Q4[#]	Q5	Q6	Q7[#]	Q8	Q9[#]	Q10	Q11[#]	Q12	Q13[#]	Q14	Q15[#]	Q16	Overall quality
Al Attar (2023)	Yes	Partial Yes	No	Partial Yes	Yes	Yes	No	Yes	Yes	No	Yes	Yes	Yes	Yes	No	Yes	Critically Low
Al Attar (2024)	Yes	Partial Yes	No	Partial Yes	Yes	No	No	Yes	Yes	No	Yes	Yes	Yes	Yes	Yes	No	Critically Low
Asplund (2009)	Yes	No	No	Partial Yes	No	Yes	No	Yes	No	No	NA	NA	No	Yes	NA	Yes	Critically Low
Benson (2009)	Yes	No	No	Partial Yes	Yes	No	No	Yes	Yes	No	NA	NA	Yes	Yes	NA	Yes	Critically Low
Chavarro-Nieto (2021)	Yes	No	No	Partial Yes	Yes	Yes	No	Yes	Yes	No	NA	NA	No	Yes	NA	Yes	Critically Low
Cusimano (2010)	Yes	No	No	Partial Yes	Yes	Yes	Yes	Yes	Yes	No	NA	NA	Yes	Yes	NA	Yes	Critically Low
Cusimano (2013)	Yes	No	No	Partial Yes	No	Yes	Yes	Yes	Yes	No	NA	NA	Yes	Yes	NA	Yes	Critically Low
Daly (2021)	Yes	No	No	Partial Yes	No	Yes	No	Yes	Yes	No	NA	NA	No	Yes	NA	Yes	Critically Low
Delgadillo (2025)	Yes	No	No	Partial Yes	Yes	Yes	No	Yes	Yes	No	NA	NA	Yes	Yes	NA	Yes	Critically Low
Eliason (2023)	Yes	Yes	No	Partial Yes	Yes	Yes	No	Yes	Yes	No	Yes	Yes	Yes	Yes	No	Yes	Critically Low
Elliott (2021)	Yes	No	No	Partial Yes	Yes	Yes	No	Yes	Yes	No	NA	NA	No	Yes	NA	Yes	Critically Low
Emery (2017)	Yes	Yes	No	Partial Yes	Yes	Yes	No	Yes	Yes	No	Yes	Yes	Yes	Yes	No	Yes	Critically Low
Enniss (2018)	Yes	Partial Yes	No	Partial Yes	Yes	Yes	No	Yes	Yes	No	NA	NA	No	Yes	NA	Yes	Critically Low
Feiss (2020)	Yes	No	No	Partial Yes	Yes	Yes	Yes	Yes	Yes	No	NA	NA	Yes	Yes	NA	Yes	Critically Low
Fraas & Burchiel (2016)	No	No	No	Partial Yes	No	No	No	No	No	No	NA	NA	Yes	No	NA	Yes	Critically Low
Ivanic (2024)	Yes	Yes	No	Partial Yes	Yes	Yes	Yes	Yes	Yes	No	Yes	Yes	Yes	Yes	Yes	Yes	Critically Low
Knapik (2019)	Yes	Partial Yes	No	Partial Yes	No	No	No	Yes	Yes	No	Yes	No	Yes	No	No	Yes	Critically Low
Knight (2021)	Yes	No	No	Partial Yes	Yes	No	No	Yes	Yes	No	NA	NA	Yes	Yes	NA	Yes	Critically Low
Pankow (2021)	Yes	Yes	No	Partial Yes	Yes	Yes	No	Yes	Yes	No	Yes	No	Yes	No	No	Yes	Critically Low
Peek (2020)	Yes	Yes	No	Partial Yes	No	Yes	No	Yes	Yes	No	NA	NA	Yes	Yes	NA	Yes	Critically Low
Rennie (2024)	No	Partial Yes	No	Partial Yes	No	No	No	No	Yes	No	NA	NA	Yes	Yes	NA	Yes	Critically Low
Tjønndal (2022)	Yes	No	No	Partial Yes	Yes	No	No	Yes	Yes	No	NA	NA	Yes	Yes	NA	Yes	Critically Low
West (2023)	Yes	Yes	No	Partial Yes	Yes	Yes	No	Yes	Yes	No	NA	NA	No	Yes	NA	Yes	Critically Low
Shill et al. (2025)	Yes	Yes	No	Partial Yes	Yes	Yes	No	Yes	Yes	No	Yes	No	No	Yes	No	Yes	Critically Low
Chen et al. (2025)	Yes	Yes	No	Partial Yes	Yes	Yes	No	Yes	Yes	No	Yes	No	No	Yes	No	Yes	Critically Low

1 = Word research question and inclusion criteria according to PICOS (population, intervention, comparator, outcome, study design), 2 = Establish methods prior to the conduct of the meta-analyses (written protocol), 3 = Explain the choice of study design for inclusion, 4 = Use comprehensive literature search strategy, 5 = Perform study selection in duplicate, 6 = Perform data extraction in duplicate, 7 = Provide a list of excluded studies to justify the exclusion, 8 = Describe the included studies in detail, 9 = Assess the risk of bias, 10 = Reported sources of funding for included studies, 11 = Use appropriate method for statistical combination of results, 12 = Assess the potential impact of risk of bias for included studies, 13 = Account for risk of bias while interpreting/discussing the results, 14 = Explain/discuss any heterogeneity, 15 = Assess publication bias and discuss its impact on the results, 16 = Report potential sources of conflict of interest and describe any funding, [#] = critical weakness, NA = not applicable.

TABLE S4. Characteristics of studies included in the overview of systematic reviews.

Study (year)	Type of review	Databases	Search period/date	Number of studies	Publication date range	Study design
Al-Attar (2023)	SR and MA	Cochrane Library, AMED, PubMed, WoS, PEDro	Between 1985 and 2023	3	2001–2020	RCT
Knapik (2019)	SR and MA	PubMed, Ovid Embase, CINAHL	Final search completed in August 2018.	5	1987–2008	Prospective cohort
Al-Attar (2024)	SR and MA	Cochrane Library, PubMed, WoS, PEDro	Between 2003 and 2023	2	2018–2023	Cluster RCT
Chen (2025)	SR and MA	PubMed, CINAHL, MEDLINE, SPORTDiscus, Scopus, and Embase	From January 2002 to March 2024	8	2015–2024	Cluster RCT (n = 3), cohort (n = 3), Quasi-experimental (n = 1), pre-post intervention (n = 1)
Ivanic (2024)	SR and MA	MEDLINE, Embase, CINAHL, Scopus, WoS CC, SPORTDiscus	Up to 26 June 2023	3	2017–2021	Cluster RCT (n = 2), Quasi-experimental (n = 1)
Shill (2025)	SR and MA	Medline ALL-OVID, CINAHL Plus with Full Text-EBSCOhost, APA PsycINFO-OVID, Cochrane Database of Systematic Reviews-OVID, CENTRAL-OVID, SPORTDiscus with Full Text, EBSCOhost, EMBASE-OVID, ERIC-EBSCOhost	Searches performed on 30 October 2023 and 23 November 2023 for CENTRAL and CDSR.	16 (females only)	2005–2022	RCT (n = 3), quasi-experimental (n = 1), cross-sectional (n = 1), cohort (n = 10), case cross-over (n = 1)
Eliason (2023)	SR and MA	MEDLINE, CINAHL, APA, PsycINFO, Cochrane (Systematic Review and Controlled Trails, Registry), SPORTDiscus, EMBASE, ERIC	October 2019 and updated in March 2022	192	2001–2022	Cohort (n = 93), Case-control (n = 35), Cross-sectional (n = 34), RCT (n = 14), Quasi-experimental (n = 12), Other (n = 4)
Pankow (2021)	SR and MA	CINAHL, Cochrane Central Register of Controlled Trials, EMBASE, ERIC, Ovid MEDLINE and Epub Ahead of Print, In-Process & Other Non-Indexed Citations and Daily, ProQuest Dissertations & Theses Global Database, PsycINFO, Scopus, SPORTDiscus	1946 to May 2020	8	2015–2019	Cohort (n = 6), Quasi-experimental (n = 1), Pre-experimental (n = 1)
West (2023)	SR and MA	Ovid MEDLINE (R) In-Process & Other Non-Indexed Citations, CINAHL (Ebsco), APA PsycINFO (OVID), Cochrane Central Register of Controlled Trials (OVID), Cochrane Database of Systematic Reviews (OVID), SPORTDiscus (Ebsco), EMBASE (OVID), ERIC (Ebsco) and the WoS, Conference Proceedings Citation Index—Science, Conference Proceedings Citation Index—Social Sciences & Humanities and Emerging Sources Citation Index.	Inception to January 3, 2022	8	1995–2021	RCT (n = 2), Prospective cohort (n = 4), Retrospective cohort (n = 2)
Asplund (2009)	SR	MEDLINE, Cochrane	Through January 2009	6	1995–2008	Prospective cohort (n = 5), Retrospective cohort (n = 1)
Benson (2009)	SR	Ovid MEDLINE, Ovid Healthstar, SportDiscus, Embase, PubMed, CINAHL, OVID OLDMEDLINE, Cochrane Database of Systematic Reviews, ACP Journal Club, Cochrane Central Register of Controlled Trials, AMED—Allied and complementary medicine	1970–2007	51	1970–2007	Mostly observational; 23 were analytical epidemiologic (cohort, case-control)
Chavarro--Nieto (2021)	SR	PubMed, SPORTDiscus, WoS, Scopus	Up to January 1, 2021	14	2005–2020	Cross-sectional (n = 6), Cohort (n = 8)
Cusimano (2010)	SR	OVID Medline, OVID HealthStar, CINAHL, Sport Discus, PubMed, Scholar's Portal Physical Education Index, Web of Science, and the Cochrane Controlled Clinical Trials Register	Up to May 2010	10	1987–2009	RCT (n = 2), Prospective Cohort (n = 2), Case-Control (n = 1), Ecological (n = 1), Case Series (n = 3), Poor-quality Cohort (n = 1)

TABLE S4. Continue

Study (year)	Type of review	Databases	Search period/date	Number of studies	Publication date range	Study design
Cusimano (2013)	SR	MEDLINE (Ovid, PubMed), Embase, CINAHL, Journals @ Scholars Portal, Cochrane Library, PsycINFO, WoS	Up to July 2012	18	up to 2012	13 rule change studies, 3 educational, 2 psychosocial interventions
Daly (2021)	SR	PubMed, CINAHL, Science Direct, and WoS	NR	3	2013–2019	Intervention studies
Delgadillo (2025)	SR	PubMed, ScienceDirect, Google Scholar	Up to June 2024	15	2016–2023	RCTs (n = 15); prospective controlled trials (n = 11)
Elliott (2021)	SR	Ovid MEDLINE, CINAHL, EMBASE, SPORTDiscus, and WoS	Until May 2020	6	2013–2019	Pre experimental study (n = 2), Cohort (n = 2), Cluster RCT (n = 2)
Emery (2017)	SR and MA	Ovid MEDLINE, CINAHL, PsycINFO, Cochrane (x3), SPORTDiscus, EMBASE, ERIC, CPCI (WoS), ProQuest Dissertations	Up to September 2016	48	NR	RCT (n = 5), cohort (n = 20), historical cohort (n = 12), case-control (n = 3), cross-sectional (n = 5), pre-experimental (n = 2), ecological (n = 1)
Enniss (2018)	SR	MEDLINE, PubMed, Scopus, Cumulative Index of Nursing and Allied Health Literature, and Embase	January 1, 1985, to November 30, 2017	31 (7 assessed with GRADE for concussion-specific outcomes)	NR	3 RCTs (methodological flaws), 28 observational (retrospective/prospective cohort)
Feiss (2020)	SR	SPORTDiscus, Academic Search Premiere, CINAHL, PsycINFO, PubMed, and Google Scholar	Search conducted December 11, 2019	3 (concussion incidence only, out of 13 total)	2014–2016	3 observational (non-randomized)
Fraas (2016)	SR	PubMed, PsycInfo, MEDLINE, SPORTDiscus, WoS	NR	10	2004–2015	Commentaries: 2 studies; Editorials: 2 studies; Literature reviews: 2 studies; Ecological/Cross-sectional: 1 study; Qualitative research: 1 study; Cohort study: 1 study; Systematic review of low-quality studies: 1 study
Knight (2021)	SR	Ovid MEDLINE, Cochrane Library, Scopus, PsycINFO and SPORTDiscus	In April 2020	8	2005–2020	4 prospective cohort injury surveillance studies, 2 RCTs, 1 pilot RCT, 1 cross-sectional survey
Peek (2020)	SR	EMBASE, MEDLINE, CINAHL, SportsDiscus and WoS	To January 2019	5	2008–2018	Cross-sectional (n = 5)
Rennie (2024)	SR	PubMed, ScienceDirect, CINAHL, MEDLINE, and SPORTDiscus	To August 2023	46	2002–2023	Observational, prospective, retrospective, cross-sectional, longitudinal, and intervention studies
Tjønndal (2022)	SR	SPORTDiscus, ERIC, PubMed, PsycINFO, and ISI WoS	Between February 2019 and May 2020	total = 39 (medical studis included = 16)	1980 and onwards	Most observational

Abbreviations: AXIS: Appraisal tool for Cross-Sectional Studies, CI: Confidence interval F: Female, GRADE: Grading of Recommendations, Assessment, Development and Evaluations, HIA: Head injury Assessment, HQ: High quality, HR: Hazard ratio, HUF: Heads Up Football, I^2: I squared (heterogeneity), IRR: Incidence rate ratio, LQ: Low quality, M: Male, MA: Meta-analysis, MQ: Moderate quality, NCI: Not clearly indicated, NMT: Neuromuscular training, NIH: National Institutes of Health Quality Assessment Tool, NS: not significant, NOS: Newcastle-Ottawa Scale, NR: Not reported, PEDro: Physiotherapy Evidence Database, RCT: Randomized controlled trial, ROBINS-I: Risk Of Bias In Non-randomized Studies – of Interventions, RoB 2: Risk of Bias 2 Tool, RR: Relative risk, SIGN: Scottish Intercollegiate Guidelines Network, SR: Systematic review, SWAT: Special weapons and tactics (elite law enforcement unit), WoS: Web of Science.

TABLE S4. Continue

Study (year)	Quality assessment tool (Results)	Country of studies	Sample size (exposure hours)	Age range (years)	Gender	Activity
Al-Attar (2023)	PEDro (2 HQ, 1 MQ)	Australia (n = 2), US (n = 1)	6311 (173,383)	< 15–20	M & F (n = 1), M (n = 1)	Soccer (n = 1), Rugby (n = 2)
Knapik (2019)	Downs and Black (2 GQ, 2 MQ, 1 LQ)	New Zealand (n = 1), South Africa (n = 1), USA (n = 2), England (n = 1)	2461 (NCI)	NCI	NCI	Rugby union (n = 3), Hockey (n = 1), Basketball (n = 1)
Al-Attar (2024)	PEDro (1 HQ, 1 MQ)	Saudi Arabia, multi-country (Europe)	2626 (19,0836)	7–13	M (n = 1), M&F (n = 1)	Soccer
Chen (2025)	NIH tool (average = 7)	UK (n = 3), USA (n = 2), Australia (n = 2), Kosovo (n = 1)	180 1,413,043	10.56–26.1	M (n = 7), M&F (n = 1)	Rugby (n = 4), soccer (n = 2), American football (n = 2)
Ivanic (2024)	RoB 2 (not serious)	England (n = 3)	4466 (NR)	25 (n = 1), 16 (n = 2)	M (n = 3)	Rugby union
Shill (2025)	Downs and Black (range: 6–21)	USA (n = 11), Australia (n = 2), Canada (n = 2), South Korea (n = 1)	NR	> 10	F only (16 stud es)	Team sports (mainly soccer and lacrosse)
Eliason (2023)	SIGN and Downs and Black. The median Downs and Black ROB assessment = 13/33 (range: 6–24). Based on SIGN criteria, 28 studies (13%) had low methodological quality. Of the remaining 192 studies, most (169/192; 88%) were of sufficient quality, with the remaining (23/196; 12%) excellent quality.	USA (n = 119), Canada (n = 32), UK (n = 10), Australia (n = 7), South Africa (n = 2), Other/ Not reported (n = 22)	NR	5–24 (52% child/ adolescent-focused studies)	M & F	American football (n = 70), Ice hockey (n = 42), Rugby (n = 29), Soccer (n = 18), Lacrosse (n = 13), multiple/ other (n = 20)
Pankow (2021)	Downs and Black checklist: 7 studies = sufficient (+), 1 study = excellent (++), 0 = low quality (−)	USA and Canada (exact numbers not stated)	NR	5–19	Male	Youth tackle football
West (2023)	Downs and Black; mean = 14/33 (SD 3). Only 2 RCTs scored > 20 (mean 23)	South Africa (n = 2), France (n = 1), UK (n = 2), Canada (n = 1), USA (n = 2)	Not pooled; varies across studies (e.g., some injury rates per 1000 h, others per 100,000/year)	Youth rugby players < 18; most between 13–18	Male	Rugby union (youth/school level)
Asplund (2009)	Not specified; independent review by 3 reviewers using a predefined methodology	USA, Canada	5186 (NR)	16–25	Male	Ice hockey
Benson (2009)	Custom epidemiologic criteria for internal/ external validity; no scoring tool used; common issues included selection bias, poor confounder measurement, and misclassification	Not consistently reported; includes USA, Canada, Europe	NR	16–30	Males and females	Multiple sports
Chavarro--Nieto (2021)	Newcastle–Ottawa Scale (NOS): 2 strong (7–10), 11 moderate (4–6), 1 weak (0–3)	UK (5), Australia (4), Ireland (2), Italy (1), New Zealand (1), South Africa (1)	1066 (NR)	NR	Male	Rugby
Cusimano (2010)	Downs and Black checklist	New Zealand (5), England (2), Australia (1), South Africa (1), Not specified (1 – McIntosh 2001)	NR	12–29	Male	Rugby

TABLE S4. Continue

Study (year)	Quality assessment tool (Results)	Country of studies	Sample size (exposure hours)	Age range (years)	Gender	Activity
Cusimano (2013)	Downs and Black checklist	Canada (13), USA (4), Both (1)	NR	NR	Mostly male; some studies did not report sex	Ice hockey
Daly (2021)	Quality Criteria Checklist for Primary Research (Academy of Nutrition and Dietetics); all 3 studies rated "neutral"	Australia, Canada, Ireland	70 (NR)	19.3–25.2	Male	Rugby (n = 2), American football (n = 1)
Delgadillo (2025)	Cochrane RoB 2; 1 high risk, others low or some concern	USA (All)	1489 (NR)	15–30	Male and female	Football, soccer, hockey, SWAT
Elliott (2021)	Downs and Black Quality Assessment Tool (57–86%)	NR	10745 (NR)	NR	Male and female	Rugby union (n = 3), American football (n = 1), multisport (n = 2)
Emery (2017)	Median Downs and Black = 14.5/33 (range 6–24)	NR	NR	Youth participants in 26 of 48 studies	Male and female	Ice hockey (n = 19), Football (n = 15), Rugby (n = 7), Soccer (n = 3), Basketball (n = 1), Skiing/Snowboarding (n = 1), Multi-sport (n = 2)
Enniss (2018)	GRADE: 7 concussion-related studies assessed: 3 low, 2 very low, 2 moderate	NR	NR	Pediatric ≤ 14, Adult amateur ≥ 15	NR	Football, Ice hockey, Soccer, Rugby
Feiss (2020)	ROBINS-I used; 3 = moderate risk	NR	839 (NR)	NR	Male and female	Football, soccer
Fraas (2016)	Strength of Recommendation Taxonomy (SORT): 8 studies = Level 3 (opinion, bench research), 2 studies = Level 2 (limited-quality cohort/SR)	NR	NR	NR	NR	Rugby
Knight (2021)	Newcastle-Ottawa Scale (NOS) for cohort studies (scores: 6–9/9); PEDro scale for RCTs (scores: 8/10); OCEBM scored as two	Australia, Ireland, USA, Canada, New Zealand	12064 (NR)	13–45	Male and female	Rugby (n = 6), Soccer (n = 2)
Peek (2020)	AXIS Quality Assessment Tool (the maximum AXIS score for each study was 17)	NR	190	15–24	67% female	Soccer
Rennie (2024)	Modified Downs and Black checklist (high risk of bias)	NR	NR	NR	NR	Australian rules football (n = 4), rugby league (n = 6), rugby union (n = 16), soccer (n = 20)
Tjønndal (2022)	NIH tool for observational studies	NR	NR	NR	Male	Olympic boxing

Abbreviations: AXIS: Appraisal tool for Cross-Sectional Studies, CI: Confidence interval F: Female, GRADE: Grading of Recommendations, Assessment, Development and Evaluations, HIA: Head injury Assessment, HQ: High quality, HR: Hazard ratio, HUF: Heads Up Football, I^2: I squared (heterogeneity), IRR: Incidence rate ratio, LQ: Low quality, M: Male, MA: Meta-analysis, MQ: Moderate quality, NCI: Not clearly indicated, NMT: Neuromuscular training, NIH: National Institutes of Health Quality Assessment Tool, NS: not significant, NOS: Newcastle-Ottawa Scale, NR: Not reported, PEDro: Physiotherapy Evidence Database, RCT: Randomized controlled trial, ROBINS-I: Risk Of Bias In Non-randomized Studies – of Interventions, RoB 2: Risk of Bias 2 Tool, RR: Relative risk, SIGN: Scottish Intercollegiate Guidelines Network, SR: Systematic review, SWAT: Special weapons and tactics (elite law enforcement unit), WoS: Web of Science.

TABLE S4. Continue

Study (year)	Preventive strategy	Outcome
Al-Attar (2023)	Headgear	IRR (users group vs. control group) = 1.03 (95% CI 0.82–1.30; P = 0.79, I^2 = 0.00%)
Knapik (2019)	Mouthguard	IRR (users vs. non-users) = 1.25 (95% CI 0.90–1.74, I^2 = 0.00%) NS
Al-Attar (2024)	FIFA 11+ Kids	IRR = 0.35; (95% CI 0.05–2.28, I^2 = 0.00%) NS
Chen (2025)	Educational and physical training programs (e.g., HUF, NMT)	All injury prevention programs: IRR = 0.66 (95% CI 0.50–0.85 I^2 = 0.00%) P = 0.002<br>IRR (physical) = 0.61 (95% CI 0.41–0.89, I^2 = 0.00%)<br>IRR (educational) = 0.76 (95% CI 0.45–1.30)<br>Educational *vs.* physical: NS
Ivanic (2024)	Neuromuscular warm-up programs	IRR = 0.69 (95% CI 0.39–1.23, I^2 = 69%), low-quality evidence
Shill (2025)	Headgear, eyewear, mouthguard, conditioning sessions, FIFA 11+, education programs, rule changes	Headgear:<br>Intent-to-treat IRR = 0.70 (95% CI 0.50–0.99, I^2 = 27.6%), very-low certainty<br>as-treated IRR = 0.61 (95% CI 0.44–0.85, I^2 = 27.6%), very-low certainty<br>Eyewear: IRR = 1.08 (95% CI 0.69–1.68), very-low certainty<br><br>No definitive conclusion for other primary prevention strategies.
Eliason (2023)	Headgear	IRR = 0.84 (95% CI 0.67–1.04, I^2 = 95.89%) NS
	Headgear	IRR (Australian football, females) = 1.09 (95% CI 0.40–95) NS
		IRR (soccer) = 0.64 (95% CI 0.44–0.92, I^2 = 38.52%) P = 0.02
		IRR (Lacrosse) = 0.24 (95% CI 0.04–1.30) NS
		IRR (rugby, lacrosse, Australian football): NS
		IRR different age groups: NS
	Mouthguard	IRR = 0.74 (95% CI 0.64–0.85, I^2 = 0.00%) P = 0.00
		IRR (ice hockey) = 0.72 (95% CI 0.60–0.87, I^2 = 9.26%) P = 0.00
		IRR (rugby) = 0.80 (95% CI 0.51–1.27, I^2 = 0.00%) NS
		IRR (adults) = 0.62 (95% CI 0.42–0.91, I^2 = 0.00%) P = 0.01
		IRR (Mix) = 0.76 (95% CI 0.65–0.89, I^2 = 0.00%) P = 0.00
		Dentist-fitted mouthguards and other specialized types vs. off-the-shelf mouthguards: equal protection
	Helmets	Results are contradictory.
	Face-shields/faceguards	Findings in ice hockey are mixed.
	Eyewear	The SRC rate increased in lacrosse players and did not change in hockey players.
	Jugular vein compression collars	Lack of data on concussion precludes conclusion.
	Policy, rule, or law changes	
	Body checking in youth ice hockey	IRR = 0.42 (95% CI 0.33–0.53, I^2 = 0.00%) P = 0.00
		IRR (adolescents) = 0.42 (95% CI 0.26–0.69, I^2 = 61.69%) P = 0.00
		IRR (children) = 0.42 (95% CI 0.31–0.59, I^2 = 0.00%) P = 0.00

TABLE S4. Continue

Study (year)	Preventive strategy	Outcome
	Policy changes in American football	IRR = 0.36 (95% CI 0.16–0.80, I^2 = 97.94%) P = 0.01
		IRR (partial equipment; males) = 0.29 (95% CI 0.25–0.33, I^2 = 0.00%) P = 0.00
		IRR (reduced contact practices; males) = 0.9 (95% CI 0.23–3.51, I^2 = 95.59%) NS
		IRR (reduced intensity; males) = 0.19 (95% CI 0.06–0.64)
		IRR (restricted collision time; sex not stated) = 0.30 (95% CI 0.09–0.98, I^2 = 80.24%) P = 0.01
		IRR (adolescents ; males) = 0.44 (95% CI 0.25–0.75) P = 0.00
		IRR (adults; males) = 0.36 (95% CI 0.14–0.93, I^2 = 80.24%) P = 0.01
		IRR (mix; sex not stated) = 0.30 (95% CI 0.09–0.98) P = 0.05
		Prior bodychecking experience did not lower concussion rates.
Eliason (2023)	Rules	Fair play rules (ice hockey, 11–14 years old):
		Reduced head impacts but not SRC rates.
		No association between team penalty minutes and opponents' SRC rates.
		Game suspension policy for excessive penalty minutes was associated with lower odds of SRC.
		Collision Rules (baseball, home plate collisions):<br>Limiting collisions at home plate reduced catcher concussion rates.
		Substitution and contact frequency rules (rugby and football):<br>Rule limiting interchange frequency in rugby league did not reduce SRC rates.<br>Lowering tackle height in rugby union did not reduce SRC rates.
		Targeting and head contact rules (Ice Hockey, Football):<br>Rule penalizing targeting the head in professional ice hockey (NHL Rule 48) was not associated with reduced SRC rates.<br>Zero tolerance for head contact in youth hockey (11–14 years) did not reduce SRC rates; rates increased after the rule.
		Targeting rules in American football showed mixed results: some studies showed SRC reductions, others did not.<br>Targeting penalties during plays in collegiate football were associated with much higher SRC rates compared to non-targeting plays.
		Pass and Obstruction Rules (Professional Ice Hockey):<br>Removal of the two-line pass rule and stricter obstruction enforcement decreased SRC rates.
		Penalty Rules (soccer, elbow to head contact):<br>Red card enforcement for elbow to head in soccer showed a non-significant reduction in SRC.<br>Heading ban (youth soccer, USA):<br>Heading ban was associated with an increase in SRC relative to other injuries.<br>Minimizing bodychecking (lacrosse):<br>Rules minimizing intentional head contact in adolescent lacrosse reduced bodychecking-related SRC during practices.
		Grouping by age and weight (youth football):<br>Grouping youth football players by age and weight instead of age only did not reduce SRC rates.
		Contact limitation policies (football practice):<br>Restricting contact in youth football practices reduced practice-related SRC rates.<br>Similar contact restrictions at the collegiate level football were not successful in reducing SRC rates.<br>Policies limiting practice contact across football leagues led to a 64% reduction in practice-related SRC rates.
		Kickoff rule changes (collegiate football):<br>Moving the kickoff line significantly reduced SRC rates on kickoffs.
	Training	Vision training and core strength programs were associated with reduced SRC rates in youth and adolescent athletes.
		NMT warm-ups reduced SRC rates when performed regularly, mainly in rugby players.
Eliason (2023)	Education	Comprehensive coach education programs and contact restrictions in practices reduced practice-related SRC rates in youth football.
		Adding a player safety coach further reduced practice-related SRCs but did not clearly impact game-related SRCs.
		Poor balance and lower weekly training hours were associated with a higher risk of SRC.

TABLE S4. Continue

Study (year)	Preventive strategy	Outcome
Pankow (2021)	Protective equipment	Two of three studies found differences in concussion rates between helmet models/brands.
		Helmets with thicker zygoma pads reduced concussion risk.
		Poor helmet fit was linked to more concussion symptoms and longer duration.
		Helmet age and reconditioning were not associated with concussion outcomes.
		Custom-made mouthguards were linked to higher concussion odds than non-custom types.
	Policy change	Concussion rates decreased after limiting contact in high school football practices.
		Restricting collision practices was associated with a lower incidence of concussion.
		NS difference in concussion rates between leagues with age-only and age-plus-weight restrictions.
	Training Interventions	HUF combined with contact restriction policies was associated with reduced concussion rates in youth football.
		High school players in non-HUF leagues had significantly higher concussion rates than those in HUF leagues.
		The presence of a player safety coach in HUF programs was linked to lower concussion rates during practices.
		Other contact training strategies showed limited or no reported effects on concussion rates.
West (2023)	Protective equipment	Mouthguards (U18): HR = 0.70 (95% CI 0.54–0.92): Significant reduction in concussion/head injuries
		Mouthguards (U15): HR = 1.69 (95% CI 0.98–2.94) NS
		Headgear: IRR = 0.95–1.13; HR = 1.23 (95% CI 0.89–1.69): NS
		Shoulder pads: HR = 1.02–1.19; NS
	NMT	NMT $\geq 3 \times$/week: RR = 0.41 (90% CI 0.17–0.99): Significant 59% reduction in concussion
		NMT any frequency: RR = 0.71 (95% CI 0.48–1.05): NS
Asplund (2009)	Protective equipment	NS difference in concussion risk between full and half face shields: RR = 0.97 (95% CI 0.61–1.54), P = 0.90
		NS difference in concussion rates across full, partial, or no facial protection.
		Players with full face shields missed fewer sessions after a concussion than those with half shields.
		Mouthguard use with full face shields was associated with no time lost post-concussion: 0 vs 1.8 sessions missed (95% CI 1.38–2.34)
Benson (2009)	Protective equipment	The effect of helmet/headgear use on concussion risk is inconclusive.
		Full facial protection in ice hockey may reduce concussion severity, as measured by time loss from competition.
		NS difference in concussion rates between full, partial, and no facial protection.
Chavarro--Nieto (2021)	Neck strengthening	No conclusion can be drawn due to the lack of studies.
Cusimano (2010)	Equipment, education, and rules	Headgear use was not effective in reducing concussion incidence in rugby.
		Mouthguards showed inconsistent results, with most studies reporting no significant reduction in concussion risk.
		Educational programs (e.g., RugbySmart) were associated with a significant reduction in concussion incidence.
		System-wide interventions combining education and rule enforcement were the most effective strategies for reducing concussions.
		Studies suggesting benefits from mouthguards had methodological limitations.
Cusimano (2013)	Rules and education	Rule changes, especially restricting bodychecking, were associated with significant reductions in concussion rates in youth ice hockey.
		Delaying the introduction of bodychecking (e.g., starting at an older age) was linked to fewer concussions.
		Educational programs reduced aggressive behavior but were not designed or powered to assess concussion outcomes.
		Cognitive-behavioral interventions showed promise in reducing aggression, but their direct effect on concussions remains unclear.
Daly (2021)	Neck strengthening	There is a lack of evidence.
Delgadillo (2025)	Jugular vein compression	There is a lack of evidence.

TABLE S4. Continue

Study (year)	Preventive strategy	Outcome
Elliott (2021)	Neck strengthening	Athletes with greater neck strength have a lower risk of sustaining SRC
		Injury reduction programs, which contain neck exercises, can reduce the incidence of related head and neck injuries, including concussion.
Emery (2017)	Body checking (ice hockey) Mouthguard (basketball, ice hockey, rugby)	IRR = 0.3 (95% CI 0.22–0.41 I^2 = 0.19%) P = 0.00
		IRR (ice hockey) = 0.81 (95% CI 0.6–1.10, I^2 = 0.00%) NS
		Helmets in skiing/snowboarding reduce the risk of concussion (based on one study)
Enniss (2018)	Equipment, education, neck strength training, and rules	Strong recommendation for head protective equipment in both pediatric and adult amateur athletes.
		Conditional support for concussion education and rules prohibiting high-risk activities.
		Conditional support for neck strengthening in adult amateur athletes.
		Strong recommendation for newer football helmet technology in adult athletes.
		Strong recommendation for rules regulating body-checking in youth ice hockey.
Feiss (2020)	Education program	Concussion education for coaches, particularly in-person training, can lead to reduced concussion risk.
Fraas (2016)	Education program for coaches/ referees	There is no data related to the effectiveness of these programs in preventing concussion.
Knight (2021)	Headgear	No consistent evidence
		5 out of 8 studies reported no difference in concussion rates between headgear users and non-users.
		One rugby study reported lower concussion risk in non-headgear users.
		One soccer study found higher concussion risk in non-headgear users.
Peek (2020)	Neck strengthening	The most effective method to strengthen the neck and reduce head acceleration during purposeful heading and the impact this has on concussion incidence in soccer is still unknown.
Rennie (2024)	Rule changes related to match structure, scoring, player safety, interchange, etc.	Lowering tackle height in rugby improved tackling behavior but did not consistently reduce concussion incidence.
		Implementation of HIA protocols increased concussion detection but did not serve as a preventive measure.
		Rule changes allowing concussion substitutions in soccer did not reduce the occurrence of head collision events.
		Penalizing intentional elbow-to-head contact in soccer helped reduce overall head injuries, but concussion-specific outcomes were unclear.
		Modifying goal kick rules in soccer may reduce head-to-head collisions, with potential indirect benefit for concussion prevention.
		Rule changes had inconsistent effects on concussion incidence.
Tjønndal (2022)	Headgear	The role of headguards in mitigating concussions in boxing remains unclear.
		Some headguards provide limited protection against linear impacts, but less so for rotational impacts.
		The removal of headguards in elite male boxing was based on behavioral assumptions.
		Lack of high-quality longitudinal studies or RCTs.
		Head injuries still occur with or without headguards.
		Future research is needed, especially among female, youth, and recreational boxers.

Abbreviations: AXIS: Appraisal tool for Cross-Sectional Studies, CI: Confidence interval F: Female, GRADE: Grading of Recommendations, Assessment, Development and Evaluations, HIA: Head injury Assessment, HQ: High quality, HR: Hazard ratio, HUF: Heads Up Football, I^2: I squared (heterogeneity), IRR: Incidence rate ratio, LQ: Low quality, M: Male, MA: Meta-analysis, MQ: Moderate quality, NCI: Not clearly indicated, NMT: Neuromuscular training, NIH: National Institutes of Health Quality Assessment Tool, NS: not significant, NOS: Newcastle-Ottawa Scale, NR: Not reported, PEDro: Physiotherapy Evidence Database, RCT: Randomized controlled trial, ROBINS-I: Risk Of Bias In Non-randomized Studies – of Interventions, RoB 2: Risk of Bias 2 Tool, RR: Relative risk, SIGN: Scottish Intercollegiate Guidelines Network, SR: Systematic review, SWAT: Special weapons and tactics (elite law enforcement unit), WoS: Web of Science.
